# Supplementary material for: Practice assistants´ perceived mental workload: A cross-sectional study with 550 German participants addressing work content, stressors, resources, and organizational structure
Source: PLoS One. 2020 Oct 1;15(10):e0240052. doi: 10.1371/journal.pone.0240052 (PMC7529273; doi:10.1371/journal.pone.0240052)
Supplement: Attachment — Submitted filename: PONE-D-20-07803_R1_reviewer.pdf [file pone.0240052.s002.pdf]

PLOS ONE

Practice assistants´ perceived mental work load: A cross-sectional study with 550 German participants addressing work content, stressors, resources, and organizational structure

--Manuscript Draft--

**Manuscript Number:**	PONE-D-20-07803R1
**Article Type:**	Research Article
**Full Title:**	Practice assistants´ perceived mental work load: A cross-sectional study with 550 German participants addressing work content, stressors, resources, and organizational structure
**Short Title:**	Practice assistants´ perceived mental work load
**Corresponding Author:**	Jan Hoffmann<br>Uniklinik Koln<br>Cologne, NRW GERMANY
**Keywords:**	practice assistants; Primary care; mental workload; psychosocial risk assessment; workplace characteristics
**Abstract:**	Introduction: Practice assistants represent a highly relevant occupational group in Germany and one of the most popular training professions in Germany. Despite this, most research in the health care sector has focused on secondary care settings, but has not addressed practice assistants in primary care. Knowledge about practice assistants' workplace-related stressors and resources is particularly scarce. This cross-sectional study addresses the mental workload of practice assistants working in primary care practices.<br>Methods: Practice assistants from a network of 185 German primary care practices were invited to participate in this cross-sectional study. The standardized `Short Questionnaire for Workplace Analysis' (German: Kurzfragebogen zur Arbeitsanalyse) was used to assess practice assistants´ mental workload. It addresses eleven workplace factors in 26 items: versatility, completeness of task, scope of action, social support, cooperation, qualitative work demands, quantitative work demands, work disruptions, workplace environment, information and participation, and benefits. Sociodemographic and work characteristics were also obtained. A descriptive analysis was performed for sociodemographic data and "Short Questionnaire for Workplace Analysis" factors. The one-sided t-test and Cohen´s d were calculated for a comparison with data from 23 professional groups (n=8,121).<br>Results: A total of 550 practice assistants from 130 practices participated. The majority of practice assistants was female (98.5%) and worked full-time (64.5%) in group practices (50.2%). Compared to the other professional groups, practice assistants reported higher values for the factor social support (4.0 versus 3.7 [d 0.44; p<0.001]), information and participation (3.6 versus 3.3 [d 0.38; p<0.001] as well as work disruptions (2.7 vs. 2.4 [d 0.42; p<0.001]), while practice assistants showed lower values regarding scope of action (3.4 versus 3.8 [d 0.43; p<0.001]).<br>Conclusions: Our study identified social support and participation within primary care practices as protective factors for mental workload, while work disruptions and scope of action were perceived as stressors.
**Order of Authors:**	Jan Hoffmann
	Chrstine Kersting
	Birgitta Weltermann
**Response to Reviewers:**	Journal Requirements:<br><br>When submitting your revision, we need you to address these additional requirements.<br>1. Please ensure that your manuscript meets PLOS ONE's style requirements, including those for file naming. The PLOS ONE style templates can be found at<br>https://journals.plos.org/plosone/s/file?id=wjVg/PLOSOne_formatting_sample_main_body.pdf and<br>https://journals.plos.org/plosone/s/file?id=ba62/PLOSOne_formatting_sample_title_aut

Powered by Editorial Manager® and ProduXion Manager® from Aries Systems Corporation

hors_affiliations.pdf

Answer: Our manuscript now meets all style requirements.

2. We suggest you thoroughly copyedit your manuscript for language usage, spelling, and grammar. If you do not know anyone who can help you do this, you may wish to consider employing a professional scientific editing service.

Whilst you may use any professional scientific editing service of your choice, PLOS has partnered with both American Journal Experts (AJE) and Editage to provide discounted services to PLOS authors. Both organizations have experience helping authors meet PLOS guidelines and can provide language editing, translation, manuscript formatting, and figure formatting to ensure your manuscript meets our submission guidelines. To take advantage of our partnership with AJE, visit the AJE website (http://learn.aje.com/plos/) for a 15% discount off AJE services. To take advantage of our partnership with Editage, visit the Editage website (www.editage.com) and enter referral code PLOSEDIT for a 15% discount off Editage services. If the PLOS editorial team finds any language issues in text that either AJE or Editage has edited, the service provider will re-edit the text for free.
Upon resubmission, please provide the following:
a) The name of the colleague or the details of the professional service that edited your manuscript
b) A copy of your manuscript showing your changes by either highlighting them or using track changes (uploaded as a *supporting information* file)
c) A clean copy of the edited manuscript (uploaded as the new *manuscript* file)

Answer: The manuscript was proofread by a professional medical translator (Sarah Chalmers; https://www.medi-translate.com/)

3. We note that you have indicated that data from this study are available upon request. PLOS only allows data to be available upon request if there are legal or ethical restrictions on sharing data publicly. For information on unacceptable data access restrictions, please see http://journals.plos.org/plosone/s/data-availability#loc-unacceptable-data-access-restrictions.
In your revised cover letter, please address the following prompts:
a) If there are ethical or legal restrictions on sharing a de-identified data set, please explain them in detail (e.g., data contain potentially identifying or sensitive patient information) and who has imposed them (e.g., an ethics committee). Please also provide contact information for a data access committee, ethics committee, or other institutional body to which data requests may be sent.
b) If there are no restrictions, please upload the minimal anonymized data set necessary to replicate your study findings as either Supporting Information files or to a stable, public repository and provide us with the relevant URLs, DOIs, or accession numbers. Please see http://www.bmj.com/content/340/bmj.c181.long for guidelines on how to de-identify and prepare clinical data for publication. For a list of acceptable repositories, please see http://journals.plos.org/plosone/s/data-availability#loc-recommended-repositories.
We will update your Data Availability statement on your behalf to reflect the information you provide.

Answer: The data cannot be shared publicly because of ethical restrictions and data protection issues as our dataset includes potentially identifying information.

4. Please ensure that you refer to Figure 1 in your text as, if accepted, production will need this reference to link the reader to the figure.

Answer: A reference to Figure 1 is now included (line 208).
“As illustrated in Fig 1, the comparison of our results with data from Nolting et al. [8] revealed statistically significant differences ($p < 0.05$) for the following factors: versatility (3.6 vs. 3.8), completeness of task (3.5 vs. 3.6), scope of action (3.4 vs. 3.8), social support (4.0 vs. 3.7), cooperation (3.6 vs. 3.4), qualitative work demands (2.2 vs. 2.1), works disruptions (2.7 vs. 2.4), information and participation (3.6 vs. 3.3), and benefits (2.9 vs. 2.4).”

5. We note you have included a table to which you do not refer in the text of your manuscript. Please ensure that you refer to Table 4 in your text; if accepted, production will need this reference to link the reader to the Table.

Answer: A reference to Table 4 is now included (line 221).

“Table 4 shows a comparison of PrAs in our study population (from 2014) and the comparative study population (from 2000).”

[Note: HTML markup is below. Please do not edit.]

Reviewers' comments:

Reviewer's Responses to Questions
Comments to the Author

1. Is the manuscript technically sound, and do the data support the conclusions?

The manuscript must describe a technically sound piece of scientific research with data that supports the conclusions. Experiments must have been conducted rigorously, with appropriate controls, replication, and sample sizes. The conclusions must be drawn appropriately based on the data presented.
Reviewer #1: Yes

2. Has the statistical analysis been performed appropriately and rigorously?
Reviewer #1: Yes

3. Have the authors made all data underlying the findings in their manuscript fully available?

The PLOS Data policy requires authors to make all data underlying the findings described in their manuscript fully available without restriction, with rare exception (please refer to the Data Availability Statement in the manuscript PDF file). The data should be provided as part of the manuscript or its supporting information, or deposited to a public repository. For example, in addition to summary statistics, the data points behind means, medians and variance measures should be available. If there are restrictions on publicly sharing data—e.g. participant privacy or use of data from a third party—those must be specified.
Reviewer #1: No

Answer: The data cannot be shared publicly because of ethical restrictions and data protection issues as our dataset includes potentially identifying information.

4. Is the manuscript presented in an intelligible fashion and written in standard English?

PLOS ONE does not copyedit accepted manuscripts, so the language in submitted articles must be clear, correct, and unambiguous. Any typographical or grammatical errors should be corrected at revision, so please note any specific errors here.
Reviewer #1: No

Answer: The manuscript was proofread by a certified medical translator.

5. Review Comments to the Author

Please use the space provided to explain your answers to the questions above. You may also include additional comments for the author, including concerns about dual publication, research ethics, or publication ethics. (Please upload your review as an attachment if it exceeds 20,000 characters)
Reviewer #1:
1. The manuscript is full of typography errors; punctuations.

Answer: The manuscript was proofread by a professional medical translator.

	2. Language is main problem<br><br>Answer: The manuscript was proofread by a professional medical translator.<br><br>3. Not consistent throughout the document<br><br>Answer: The manuscript was proofread by a professional medical translator.<br><br>4. Don’t use abbreviation in the abstract part<br><br>Answer: This was corrected.<br><br>5. In the background part there are incomplete sentences<br><br>Answer: This was corrected.<br><br>6. In tables the decimal places should be consistent<br><br>Answer: This was corrected in Tables 1, 2,3 and 4.<br><br>7. In the table reporting missing value is not necessary<br><br>Answer: This was corrected in Tables 1 and 2.<br><br>6. PLOS authors have the option to publish the peer review history of their article (what does this mean?). If published, this will include your full peer review and any attached files.<br><br>If you choose “no”, your identity will remain anonymous but your review may still be made public.<br><br>Do you want your identity to be public for this peer review? For information about this choice, including consent withdrawal, please see our Privacy Policy.<br>Reviewer #1: No<br><br>Answer to reviewer comment concerning response rate<br><br>Reviewer comment: “Statistically how it can be generalized with around 30% non-response rate?<br><br>Answer: The argument in our sentence was incorrect. The total study had a response rate of 70% of practices. Within the practices, nearly all physicians and practice assistants participated indicating a high interest in the topic.<br><br>The text was revised to: It is a strength of our study that it was based on a data set with a large number of participants (550 PrAs). Also, prior analyses had shown that the practice network from which this sample was taken is representative for German primary care practices.
**Additional Information:**	
**Question**	**Response**
**Financial Disclosure**<br><br>Enter a financial disclosure statement that describes the sources of funding for the work included in this submission. Review the submission guidelines for detailed requirements. View published research articles from *PLOS ONE* for specific	Funding for this study was provided by the Ministry of Culture and Science, North-Rhine Westphalia, Germany, formerly the Ministry of Innovation, Science and Research, North-Rhine Westphalia, Germany. https://www.mkw.nrw/. The funders had no role in study design, data collection and analysis, decision to publish, or preparation of the manuscript.

examples.<br><br>This statement is required for submission and **will appear in the published article** if the submission is accepted. Please make sure it is accurate.<br><br>**Unfunded studies**<br>Enter: *The author(s) received no specific funding for this work.*<br><br>**Funded studies**<br>Enter a statement with the following details:<br>• Initials of the authors who received each award<br>• Grant numbers awarded to each author<br>• The full name of each funder<br>• URL of each funder website<br>• Did the sponsors or funders play any role in the study design, data collection and analysis, decision to publish, or preparation of the manuscript?<br>• **NO** - Include this sentence at the end of your statement: *The funders had no role in study design, data collection and analysis, decision to publish, or preparation of the manuscript.*<br>• **YES** - Specify the role(s) played.<br><br>* typeset	
**Competing Interests**<br><br>Use the instructions below to enter a competing interest statement for this submission. On behalf of all authors, disclose any competing interests that could be perceived to bias this work—acknowledging all financial support and any other relevant financial or non-financial competing interests.<br><br>This statement **will appear in the published article** if the submission is accepted. Please make sure it is accurate. View published research articles from *PLOS ONE* for specific examples.	The authors have declared that no competing interests exist.

**NO authors have competing interests**<br><br>Enter: *The authors have declared that no competing interests exist.*<br><br>**Authors with competing interests**<br><br>Enter competing interest details beginning with this statement:<br><br>*I have read the journal's policy and the authors of this manuscript have the following competing interests: [insert competing interests here]*<br><br>* typeset	
**Ethics Statement**<br><br>Enter an ethics statement for this submission. This statement is required if the study involved:<br><br>• Human participants<br>• Human specimens or tissue<br>• Vertebrate animals or cephalopods<br>• Vertebrate embryos or tissues<br>• Field research<br><br>Write "N/A" if the submission does not require an ethics statement.<br><br>General guidance is provided below. Consult the submission guidelines for detailed instructions. **Make sure that all information entered here is included in the Methods section of the manuscript.**	Ethical approval had been obtained from the Ethics Committee of the Medical Faculty of the University of Duisburg-Essen (reference number: 13-5536-BO, date of approval: 24/11/2014). All participants received written information and signed informed consent forms.

**Format for specific study types**<br><br>**Human Subject Research (involving human participants and/or tissue)**<br>• Give the name of the institutional review board or ethics committee that approved the study<br>• Include the approval number and/or a statement indicating approval of this research<br>• Indicate the form of consent obtained (written/oral) or the reason that consent was not obtained (e.g. the data were analyzed anonymously)<br><br>**Animal Research (involving vertebrate animals, embryos or tissues)**<br>• Provide the name of the Institutional Animal Care and Use Committee (IACUC) or other relevant ethics board that reviewed the study protocol, and indicate whether they approved this research or granted a formal waiver of ethical approval<br>• Include an approval number if one was obtained<br>• If the study involved *non-human primates*, add *additional details* about animal welfare and steps taken to ameliorate suffering<br>• If anesthesia, euthanasia, or any kind of animal sacrifice is part of the study, include briefly which substances and/or methods were applied<br><br>**Field Research**<br><br>Include the following details if this study involves the collection of plant, animal, or other materials from a natural setting:<br>• Field permit number<br>• Name of the institution or relevant body that granted permission	
**Data Availability**<br><br>Authors are required to make all data underlying the findings described fully available, without restriction, and from the time of publication. PLOS allows rare exceptions to address legal and ethical concerns. See the PLOS Data Policy and FAQ for detailed information.	No - some restrictions will apply

Powered by Editorial Manager® and ProduXion Manager® from Aries Systems Corporation

A Data Availability Statement describing where the data can be found is required at submission. Your answers to this question constitute the Data Availability Statement and **will be published in the article**, if accepted.<br><br>**Important:** Stating 'data available on request from the author' is not sufficient. If your data are only available upon request, select 'No' for the first question and explain your exceptional situation in the text box.<br><br>Do the authors confirm that all data underlying the findings described in their manuscript are fully available without restriction?	
**Describe where the data may be found in full sentences. If you are copying our sample text, replace any instances of XXX with the appropriate details.**<br><br>• If the data are **held or will be held in a public repository**, include URLs, accession numbers or DOIs. If this information will only be available after acceptance, indicate this by ticking the box below. For example: *All XXX files are available from the XXX database (accession number(s) XXX, XXX.*).<br>• If the data are all contained **within the manuscript and/or Supporting Information files**, enter the following: *All relevant data are within the manuscript and its Supporting Information files.*<br>• If neither of these applies but you are able to provide **details of access elsewhere**, with or without limitations, please do so. For example:<br><br>*Data cannot be shared publicly because of [XXX]. Data are available from the XXX Institutional Data Access / Ethics Committee (contact via XXX) for researchers who meet the criteria for access to confidential data.*<br><br>*The data underlying the results presented in the study are available from (include the name of the third party*	Data cannot be shared publicly because Ethical restrictions have been imposed on the data underlying this study by the Ethics Committee of the Medical Faculty of the University of Duisburg-Essen in order to protect participant confidentiality. However, requests for an ethically compliant dataset may be made to the Ethics Committee of the Medical Faculty of the Universtiy of Duisburg-Essen (ethikkommission@uk-essen.de).

and contact information or URL).<br>• This text is appropriate if the data are owned by a third party and authors do not have permission to share the data.<br><br>* typeset	
Additional data availability information:	

Manuscript Click here to access/download;Manuscript;Manuscript.docx

Practice assistants´ perceived mental workload: A cross-sectional study with 550 German participants addressing work content, stressors, resources, and organizational structure

Jan Hoffmann[1, #a*], Christine Kersting[2, #b], Birgitta Weltermann[1]

[1] Institute of General Practice and Family Medicine, University Hospital of Bonn, Bonn, Germany

[2] Institute for General Medicine, University Hospital Essen, University of Duisburg-Essen, Essen, Germany

[#a] Current Address: Institute of Medical Sociology, Health Services Research and Rehabilitation Science, University of Cologne, Cologne, Germany

[#b] Current Address: Chair for Innovation and Collaboration in Ambulatory Health Care, Faculty of Health/School of Medicine, Witten/Herdecke University, Witten, Germany

* Corresponding author

E-mail: jan.hoffmann@uk-koeln.de (JH)

Abstract

Introduction: Practice assistants represent a highly relevant occupational group in Germany and one of the most popular training professions in Germany. Despite this, most research in the health care sector has focused on secondary care settings, but has not addressed practice assistants in primary care. Knowledge about practice assistants' workplace-related stressors and resources is particularly scarce. This cross-sectional study addresses the mental workload of practice assistants working in primary care practices.

Methods: Practice assistants from a network of 185 German primary care practices were invited to participate in this cross-sectional study. The standardized `Short Questionnaire for Workplace Analysis' (German: Kurzfragebogen zur Arbeitsanalyse) was used to assess practice assistants´ mental workload. It addresses eleven workplace factors in 26 items: versatility, completeness of task, scope of action, social support, cooperation, qualitative work demands, quantitative work demands, work disruptions, workplace environment, information and participation, and benefits. Sociodemographic and work characteristics were also obtained. A descriptive analysis was performed for sociodemographic data and "Short Questionnaire for Workplace Analysis" factors. The one-sided t-test and Cohen´s d were calculated for a comparison with data from 23 professional groups (n=8,121).

Results: A total of 550 practice assistants from 130 practices participated. The majority of practice assistants was female (98.5%) and worked full-time (64.5%) in group practices (50.2%). Compared to the other professional groups, practice assistants reported higher values for the factor social support (4.0 versus 3.7 [d 0.44; p<0.001]), information and participation (3.6 versus 3.3 [d 0.38; p<0.001] as well as work disruptions (2.7 vs. 2.4 [d 0.42; p<0.001]), while practice assistants showed lower values regarding scope of action (3.4 versus 3.8 [d 0.43; p<0.001]).

Conclusions: Our study identified social support and participation within primary care practices as protective factors for mental workload, while work disruptions and scope of action were perceived as stressors.

Keywords: practice assistants, primary care, mental workload, psychosocial risk assessment, workplace characteristics

Introduction

Practice assistants (PrAs) represent the largest group of employees in the German outpatient health care sector [1] and the second most popular training profession among German women [2]. However, little is known about how PrAs perceive their work conditions. More specifically, data on the relationship between work and psychological stress in PrAs are lacking. While psychosocial assessment studies of health personnel in secondary care have been performed [3–6], only few have addressed this issue in PrAs in German primary care [1,7,8]. Therefore, it is important to further investigate PrAs' perceived level of psychological stress, as psychological strain may not only threaten PrAs' health with potentially tremendous economic costs, but may also impair high-quality patient care [9].

In recent years, increasing attention has been devoted to employees' mental health. A systematic review by Theorell et al. highlighted that job strain has an impact on the development of depressive symptoms [10]. Also, the socio-economic implications are increasingly evident: preceded only by musculoskeletal diseases, mental health conditions rank second with 16.7% of all sick leaves among German employees [11] and caused a damage of 21.7 billion Euros gross added value in 2017 [11].

The stress-strain model developed by Rohmert and Rutenfranz in 1975 differentiates between the terms 'psychological stress' and 'psychological strain'. 'Psychological stress' describes all external factors that influence one's psychological well-being. When referring to psychological stress in a work environment, the term `mental workload´ refers to employees´ exposure to individual work demands and the environment at work [12]. However, the term does not necessarily have a negative connotation [13]. 'Psychological strain' can be understood as an individual´s response to psychological stress. Thus, the same level of psychological stress may elicit a different level of psychological strain depending on an employee´s coping strategy and constitution [14]. A well-balanced amount of psychological strain can lead to a healthy and productive workflow [12], while an extreme level of

psychological strain may threaten employees' health. Studies have shown a negative association between high levels of psychological strain and mental illness [15,16].

Since 2014, the German Safety and Health at Work Act (ArbSchG) obliges employers to perform a general risk assessment of their employees' work conditions [17]. Assessing the mental workload (a so-called `psychosocial risk assessment´) is part of this risk assessment. Based on the results, employers must take countermeasures as necessary to enhance their employees' health [18]. Due to differences in work demands, work hazards, and work environments across professions there is no gold standard that defines what instrument should be used for the psychosocial risk assessment. While different instruments exist [19], the so-called Kurzfragebogen zur Arbeitsanalyse (KFZA; English: Short Questionnaire for Workplace Analysis), a questionnaire addressing perceived workload, is widely used across professions [20]. Data from more than 8,000 participants from 23 professions are available [8].

The aims of this cross-sectional study are threefold: i) to assess the mental workload of PrAs working in German primary care practices, ii) to identify resources and stressors, and iii) to compare results with aggregated data from 23 different professions.

Material and Methods

Study design and recruitment of participants

The psychosocial assessment of PrAs reported in this paper was obtained as part of a larger cross-sectional study investigating multiple aspects of stress in primary care practices. Details of the study are reported elsewhere [21,22]. Briefly, general practitioners (GPs) and PrAs of the 185 general medicine practices of the practice network of the Institute for General Medicine, University Hospital Essen, Essen, Germany, were asked to participate in the study. The practices were located in urban and rural regions of North Rhine-Westphalia (Western Germany) with an average distance of 30 km (range: 2±180 km) to the Institute. In a prior study it was shown that the practices affiliated with the network are representative for German primary care practices [23]. Practices had been invited by mail

and contacted by phone for further recruitment. Those refusing to participate were asked to answer a short questionnaire on practice characteristics and to provide reasons for non-participation. Data were collected between April and September 2014 during on-site visits. Within each practice, all GPs (practice owners and employed physicians) and PrAs including medical secretaries and PrA trainees were eligible for participation and received the study documents. The study documents comprised a study information sheet, an informed consent form to be completed by all participants, and a set of questionnaires which included sociodemographic questions and the KFZA analyzed in this paper. To ensure data protection, participants were asked to seal the completed questionnaire in an envelope. As an incentive, practice teams received a department store chain voucher of 5 euros per person, irrespective of the participation of individual team members. In addition, the dataset contained information about the practices´ location from the practice network´s database and matched with public regional data for the population size in 2012 (www.it.nrw.de). This paper follows the STROBE recommendations for reporting cross-sectional studies [24].

Ethical approval had been obtained from the Ethics Committee of the Medical Faculty of the University of Duisburg-Essen (reference number: 13-5536-BO, date of approval: 24/11/2014). All participants received written information and signed informed consent forms.

Study instrument to assess mental workload

The KFZA was developed by Prümper et al. in 1995 and is as a widely accepted screening tool for psychological stress at the workplace [25]. The questionnaire is a standardized instrument with closed questions. It is completed by the employees themselves and thus provides a subjective view of each individual’s perception of the work environment. According to DIN EN ISO 10075 “Ergonomic principles related to mental workload“, the instrument is categorized as a “precision level 2 process for overview purposes” [26]. The instrument is listed in the toolbox for “Instruments for recording mental loads” of the Federal Institute for Occupational Safety and Health and covers multiple aspects of the work environment [27]. It includes four dimensions: *work content, resources, stressors, and organizational culture.* Dimensions consist of 11 factors which are derived from 26 single items with answer options

on a Likert scale ranging from 1 (does not apply at all) to 5 (is completely true). *Work content* contains two factors (versatility, completeness of task) and five single items (learning new skills, use of knowledge, skills and ability, variety of tasks, visibility of task accomplishment, completeness of product). *Resources* contains three factors (scope of action, social support, cooperation) and nine single items (influence on sequence of activities, influence on work content, influence on workload and procedures, social support by co-workers, social support by supervisors, social cohesion within the department, necessity of cooperation, opportunity for social exchange with co-workers, feedback from supervisors and co-workers). *Stressors* contains four factors (qualitative work demands, quantitative work demands, work disruptions, workplace environment) and eight single items (excessive complexity of tasks, excessive demands on concentration, frequent work under time pressure, too much work to do, lack of information, work materials or equipment, interruptions of workflow, unfavorable physicochemical conditions, insufficient workspace and equipment). *Organizational culture* contains two factors (information and participation, benefits) and four single items (information about organizational developments, consideration of employee input, continuous education, opportunities for advancement). The dimensions job content, resources, and organizational culture represent positive aspects, and high scores are considered positive. High scores in the stressors dimension are considered negative work aspects.

Given the time constraints in primary care practices, the KFZA was deemed suitable as it takes only 10 minutes to complete. Also, data from more than 8,000 participants from 23 other professional groups are available for comparison [25]. The questionnaire can be applied throughout all professions and workspaces and is readily available for academic use [28].

Comparative data from 23 professional groups

In 2000, the Employers' Liability Insurance Association for Medical Services and Welfare Work (BGW) in cooperation with the German Employees' Health Insurance (DAK) conducted a cross-sectional study to measure stress at work [8]. A purposive sample of 27,584 employees from 23 professional groups

was selected from the BGW and DAK register: physicians, assistant pharmacists, pharmacists, office workers, teacher, hairdressers, pest controllers, alternative practitioners, unskilled laborers, kindergarten teachers, chefs, nurses, masseurs, medical laboratory technicians, porters, facility cleaners, social workers, PrAs, veterinarians, care workers for persons at risk, employees of dialysis centers, and employees of workshops for the disabled. A total of 8,121 employees participated in the study in the context of a project called `Prevention of work-related health hazards'. The KFZA was used within the scope of the study. We performed two comparative analyses using published data of the survey: first, we compared KFZA results from the study of the 23 professional groups with results from our population. Second, we compared the results for the subpopulation of PrAs from the study with results from our population. The latter comparison is particularly interesting, as it provides a longitudinal approach (data from 2000 and 2014) in a situation where the vocational training was meanwhile been revised and PrAs in Germany are professionalizing.

Data analysis

The analysis was performed using IBM SPSS Statistics for Windows, Version 25 (Armonk, NY: IBM Corp.). Data of all PrAs were analyzed. Missing data are reported for all items. Non-plausible values were recoded as missing values.

Sociodemographic and work-related characteristics were analyzed descriptively. The mean, standard deviation (SD), median, and range are reported for metric sociodemographic and work variables. The practices' population size was categorized into rural, small, medium-sized, and big cities following categorization schemes of the Federal Institute for Research on Building, Urban Affairs and Spatial Development (rural ≤ 4,999 inhabitants, small city 5000-19,999, medium-sized city 20,000-99,9999, big city ≥ 100,000).

Following Prümper et al., the results of the KFZA were evaluated by computing mean values on a factor level [25,29]: As a first overview, positive items <3 and negative items >3 are interpreted as high levels of psychological stress and indicate a need for more detailed analyses. In addition, the comparison

with data from other professional groups or from the same professional group provides information on how to set a benchmark against other results [29]. Differences between the means of our population and the comparative population were analyzed using a one-sided t-test (95% significance level; 0.05 = alpha). Additionally, Cohen´s d was calculated to estimate the effect size. 95% confidence intervals (CI) were calculated for factors of the 2014 PrA population.

Results

Study characteristics

[illegible] PrAs participated in the study (response rate 70.3%; n=130 practices). There were four implausible values that were recoded as missing values. The sociodemographic characteristics of the participants are presented in Table 1. PrAs had a mean age of 37.97 years (SD: 12.63), with 98.55% of PrAs being female. The majority of PrAs was married (50.36%), worked full-time (64.55%) on a permanent contract (84.55%) with a median work experience of 18 years (range: 0-49 years). Most (61.45%) PrAs worked 20-39 hours a week, while 24.91% of PrAs worked more than 39 hours. Most PrAs (90.73%) had completed a three-year vocational training as “Medizinische Fachangestellte” or “Arzthelferin” which combines practical training (3 days per week) and vocational training (2 days per week). Eleven percent had other backgrounds (i.e.: secretary, practice aid, other practice employee). Almost all PrAs had completed some sort of additional training: 22.4% of PrAs had completed additional training as VERAHs (106 hours of theoretical and 94 hours of practical training) or EVAs (170 to 220 hours of theoretical training and 20 to 50 hours of practical training depending on prior work experience) that allows PrAs to perform additional tasks (e.g.: home visits). On average, PrAs worked in practices with 2.96 (SD 2.15) physicians and 7.73 (SD 7.64) PrAs. Half of the practices (50.18%) were group practices. The smallest proportion of PrAs worked in practices with a low patient load per quarter (5.45%, 501-1000 patients per quarter), while the largest proportion of PrAs worked in practices with a high patient load per quarter (27.27%, >3001 patients per quarter). PrAs’ work setting characteristics are presented in Table 2.

Table 1. Practice assistants´ sociodemographic and professional training characteristics (n=550).

Variable	Total (n=550)	100%*
**[illegible]e (n=550, years)**		
[Mean (SD)]	37.97	[illegible].63)
[Median (min-max)]	38	(16-71)
**Gender [illegible])**		
Female	542	98.55
Male	4	0.73
**Marital status (n, %)**		
Single	218	39.63
Married	277	50.36
Divorced	45	8.18
Widowed	7	1.27
**Status of employment (n, %)**		
Full-time	355	64.55
Part-time	179	32.55
**Mode of employment (n, %)**		
Fixed-term	56	10.18
Permanent	465	84.55
**Working hours per week (n, %)**		
0-19	65	11.82
20-39	338	61.45
40-59	127	23.09
>60	10	1.82
**Work experience (n=550, years)**		
[Mean (SD)]	18.74	(12,46)
[Median (Min-Max)]	18	(0-49)
**PrA in training (n, %)**		

Yes	49	8.91
No	499	90.73
**Year of training (n=51, %)**		
First year	16	31.37
Second year	19	37.25
Third year	12	23.53
**Vocational training [1] (n, %)**		
Practice assistants	490	89.09
Secretary	12	2.18
Practice aid[2]	6	1.09
[illegible]er practice employees[2]	16	2.91
Other	75	13.64
**Additional training (n=137, %)**		
VERAH	14	10.22
EVA	3	2.19
VERAH/EVA + other	8	5.84
Other	105	76.64

[1] multiple answers possible, [2] no vocational training, [illegible]mbers do not add up to 100% due to missing values

Table 2. Practice assistants' work setting characteristics (n=550).

Variable	Total (n=550)	100%
**Type of practice (n, %)**		
Solo practice	147	26.73
Group practice	276	50.18
Others	122	22.18
**Number of patients per quarter (n, %)**		
501-1000	30	5.45

1001-1500	116	21.09
1501-2000	100	18.18
2001-2500	79	14.36
2501-3000	62	11.27
>3001	150	27.27
**Location of practice[1] (n, %)**		
Small city	33	6.00
Medium-sized city	128	23.27
Big city	371	67.45
**Number of physicians in practice**		
[Mean (SD)]	*2.96*	*(2.15)*
[Median (Min-Max)]	*2*	*(1-10)*
**Number of PrAs in practice**		
[Mean (SD)]	*7.73*	*(7.64)*
[Median (Min-Max)]	*5*	*(0-35)*

[1] based on 2012 number of inhabitants, [illegible]mbers do not add up to 100% due to missing values

Comparison of practice assistants with other professional groups (comparative data)

Table 3 shows the results of the KFZA analysis for PrAs and for the comparative population. For a first overview of only results from our study population, the calculation of mean values for the factor-level analysis yielded a critical score for the factor benefits (2.86 [SD 1.05]). In contrast, social support showed the highest positive factor (4.05 [SD 0.79]).

As illustrated in Fig 1, the comparison of our results with data from Nolting et al. [8] revealed statistically significant differences ($p < 0.05$) for the following factors: versatility (3.6 vs. 3.8), completeness of task (3.5 vs. 3.6), scope of action (3.4 vs. 3.8), social support (4.0 vs. 3.7), cooperation (3.6 vs. 3.4), qualitative work demands (2.2 vs. 2.1), work disruptions (2.7 vs. 2.4), information and

participation (3.6 vs. 3.3), and benefits (2.9 vs. 2.4). The two factors workplace environment (2.2 vs. 2.2) and quantitative work demands (2.9 vs. 3.0) were found to be non-significant.

Effect size showed the strongest difference for the factors social support (4.0 vs 3.7 [d 0.44]), scope of action (3.4 vs. 3.8 [d 0.43]), and benefits (2.9 vs. 2.4 [d 0.43]). The scores for social support and benefits were higher in the PrA population than in the comparative group, whereas scope of action yielded lower scores. The factor benefits, on the other hand, was critically low in both populations. The difference in work disruptions (2.7 vs. 2.4 [d 0.41]) presented a moderate effect size. The score for work disruptions was higher in the PrA population compared to the population from Nolting et al. [8].

Comparison of practice assistants from 2000 and 2014

Table 4 shows the comparison between PrAs in our study population (from 2014) and the comparative study population (from 2000). The comparison yielded statistically significant differences ($p < 0.05$) for the factors completeness of task (3.5 vs. 3.2), social support (4.0 vs. 3.9), cooperation (3.6 vs. 3.5), qualitative work demands (2.2 vs. 2.0), quantitative work demands (2.9 vs. 2.8), work disruptions (2.7 vs. 2.5), workplace environment (2.2 vs. 2.0), information and participation (3.6 vs. 3.5), and benefits (2.9 vs 2.2).

Effect size showed no effect for versatility (d 0.05), scope of action (d 0.01), social support (d 0.19), cooperation (d 0.13), quantitative work demands (d 0.12), as well as information and participation (d 0.16). A small effect size was shown for completeness of task (d 0.32), qualitative work demands (d 0.25), work disruptions (d 0.29), and workplace environment (d 0.21). The difference in the factor benefits presented a moderate effect size (d 0.62).

Table 3. KFZA results from our study of practice assistants (n=550) in comparison with comparative data from 23 professional groups (n= 8.121).

Work aspects	KFZA factor	Our study Mean score (PrAs)	95% CI	Comparison: Mean score (Nolting et al.)	Cohen´s d	P-value **
**Job content[1]**	**Versatility**	**3.6**	3.58 - 3.70	3.8	0.23	< 0.001
	**Completeness of task**	**3.5**	3.41 - 3.57	3.6	0.12	0.0045
**Resources[1]**	**Scope of action**	**3.4**	3.37 - 3.49	3.8	0.43	< 0.001
	**Social support**	**4.0**	3.98 - 4.12	3.7	0.44	< 0.001
	**Cooperation**	**3.6**	3.53 - 3.66	3.4	0.24	< 0.001
**Stressors[2]**	**Qualitative work demands**	**2.2**	2.14 - 2.29	2.1	0.13	0.0025
	**Quantitative work demands**	**2.9**	2.83 - 3.01	3.0	0.07	0.0797
	**Work disruptions**	**2.7**	2.67 - 2.81	2.4	0.41	< 0.001
	**Workplace environment**	**2.2**	2.13 - 2.30	2.2	0.02	0.7109
**Organizational culture[1]**	**Information and participation**	**3.6**	3.57 - 3.73	3.3	0.38	< 0.001
	**Benefits**	**2.9***	2.77 - 2.94	2.4*	0.43	< 0.001

[1] High scores (>3) are considered positive, [2] high scores (>3) are considered negative, * critical values ** based on a one-sided t-test comparing mean values of PrAs and Nolting et al. on a 95% significance level

Fig 1. KFZA results on a factor level divided into resources and stressors in comparison with comparative data from Nolting et al. [1] High scores (>3) are considered positive, [2] high scores (>3) are considered negative.

Table 4. KFZA factor-level comparison of PrAs from our study (n=550; year 2014) and PrAs from Nolting et al. (n=324; year 2000).

**Work aspects**	**KFZA factor**	**Our study Mean score (PrAs)**	95% CI	PrAs' results from 2000 Mean score (PrAs; Nolting et al.)	Cohen´s d	P-value
**Job content[1]**	**Versatility**	**3.6**	3.58 - 3.70	3.6	0.05	0.238
	**Completeness of task**	**3.5**	3.41 - 3.57	3.2	0.32	< 0.001
**Resources[1]**	**Scope of action**	**3.4**	3.37 - 3.49	3.4	0.01	0.765
	**Social support**	**4.0**	3.98 - 4.12	3.9	0.19	< 0.001
	**Cooperation**	**3.6**	3.53 - 3.66	3.5	0.13	0.006
**Stressors[2]**	**Qualitative work demands**	**2.2**	2.14 - 2.29	2.0	0.25	< 0.001
	**Quantitative work demands**	**2.9**	2.83 - 3.01	2.8	0.12	0.007
	**Work disruptions**	**2.7**	2.67 - 2.81	2.5	0.29	< 0.001
	**Workplace environment**	**2.2**	2.13 - 2.30	2.0	0.21	< 0.001

**Organizational**	**Information and participation**	**3.6**	3.57 - 3.73	3.5	0.16	0.002
**culture[1]**	**Benefits**	**2.9***	2.77 – 2.94	2.2*	0.62	< 0.001

[1] High scores (>3) are considered positive, [2] high scores (>3) are considered negative, * critical values ** based on a one-sided t-test comparing mean values of PrAs and Nolting et al. on a 95% significance level

Discussion

Our study identified social support within primary care practices as a resource and a protective factor for mental workload among PrAs, while the lack of benefits at work was perceived as a stressor.

When comparing data on PrAs with the aggregated data of other professional groups, we were able to perform a more informative analysis yielding slightly different results. Scope of action and work disruptions showed the largest negative difference and the strongest effect size, whereas social support and benefits showed the largest positive difference and the strongest effect size. Interestingly, when comparing with other professional groups, the factor benefits that was identified as a stressor in the single evaluation turned out to be a resource. Since the scores are rather low in both samples, lack of benefits at work might be a general problem, while PrAs might experience more benefits at work than other professional groups. PrAs in general practices tend to be responsible for a wide range of tasks in different workplaces throughout the practices, as they are the first point of contact for patients with unexpected events occurring on a regular basis [1]. This job profile may explain the high scores for work disruptions. Although PrAs are responsible for a wide range of tasks, GPs remain the decision makers, resulting in a setting-immanent limited scope of action for PrAs.

The comparison between the PrA groups from 2000 to 2014 revealed significant differences for most factors, but small effect sizes. The factor benefits showed a moderate effect size in favor of the 2014 study population. All factors, positive factors and negative factors alike, were slightly higher in our population of PrAs compared to the 2000 PrA population from Nolting et al. The increase in benefits at work and completeness of task from 2000 to 2014 may be explained by the further training opportunities for PrAs that were introduced during that time period (i.e., VERAH, EVA). Among other changes, these trainings have enabled PrAs to carry out more complex work processes autonomously (e.g.: patient education on diabetes). Additionally, they are rewarded with a better salary. Both may be signs of professionalization. In a recent study by Vu-Eickmann et al., PrAs reported a high patient

volume, which in addition to handling many tasks at once may explain the high score for work disruptions [1].

Social support is an important resource and can positively influence job satisfaction, as shown in a recent study with Portuguese nursing staff [30]. Job satisfaction was again shown to positively correlate with patient satisfaction [31]. A systematic review yielded a similar result linking social support with staff well-being in emergency departments [32]. In contrast, studies have shown that negative work aspect (i.e.: lack of benefits, limited scope of action) cause psychological strain and can lead to a higher turnover rate and depressive symptoms [10,33].

In agreement with three other studies on this topic, we showed that PrAs in primary care practices receive high social support and have a rather limited scope of action and still insufficient benefits at work [1,7,8].

Strengths and limitations

It is a strength of our study that it was based on a data set with a large number of participants (550 PrAs). Also, prior analyses had shown that the practice network from which this sample was taken is representative for German primary care practices [23]. Each participant received an incentive in the form of a 5-Euro voucher to avoid a selection bias by selecting only highly motivated PrAs. As the network is located in a rather densely populated area, our results may overrepresent PrAs working in urban areas. The KFZA proved to be a cost-effective screening tool to gain first insights into employees' psychological stressors and resources. To our knowledge this is the first study comparing PrAs' data from a psychological risk assessment in primary care with a large sample from other professions.

In our study we were only able to assess the current situation and not the state desired by PrAs, which could have provided even more insights. The comparison with data from 23 professional groups was limited as only aggregated mean results were available without standard deviations. Due to this, we were unable to calculate confidence intervals for both populations. A strength of our study is the

comparison of the results of the 2000 with the 2014 study from the same professional group. However, the PrA populations were not identical, and caution is advised when interpreting the results.

Conclusions

Mental well-being has a tremendous impact on preserving a healthy and productive workforce. Therefore, our goal must be to first identify risk factors for mental well-being at work and put them into perspective with other occupations, which we aimed to do in this study. Second, we need to develop measures to tackle risk factors for psychological strain at work and enhance protective factors such as social support, scope of action, benefits at work, and cooperation. Last, measures need to be evaluated and implemented in the everyday working life of PrAs.

List of abbreviations

CI: confidence interval; GP: general practitioner; KFZA: Kurzfragebogen zur Arbeitsanalyse (English: Short Questionnaire for Workplace Analysis); PrA: practice assistant; SD: standard deviation

Acknowledgements

We thank the Institute for General Medicine, University Hospital Essen, for supporting the conceptualization of the questionnaire, the data collection, and the provision of the data for this analysis.

References

1. Vu-Eickmann P, Loerbroks A. Psychosoziale Arbeitsbedingungen Medizinischer Fachangestellter: Ergebnisse einer qualitativen Studie zu den berufsspezifischen Belastungen, Ressourcen, Präventionsmöglichkeiten und Interventionsbedürfnissen. Z Evid Fortbild Qual Gesundhwes. 2017; 126: 43–51. doi: 10.1016/j.zefq.2017.06.005.
2. Statistisches Bundesamt. Auszubildene. nach Ausbildungsberufen 2017 (TOP 20), Frauen. Available: https://www.destatis.de/DE/ZahlenFakten/GesellschaftStaat/BildungForschungKultur/BeruflicheBildung/Tabellen/AzubiRangliste.html. Accessed 12 March 2019.
3. Freimann T, Merisalu E. Work-related psychosocial risk factors and mental health problems amongst nurses at a university hospital in Estonia: a cross-sectional study. Scand J Public Health. 2015; 43: 447–452. doi: 10.1177/1403494815579477.

4. Kern M, Buia A, Tonus C, Weigel TF, Dittmar R, Hanisch E, et al. Psychological stressors, resources and well-being of surgeons in Germany : A cross-sectional study. Chirurg. 2019. doi: 10.1007/s00104-018-0780-5.
5. Ulusoy N, Wirth T, Lincke H-J, Nienhaus A, Schablon A. Psychosocial burden and strains in geriatric nursing: comparison of nursing personnel with and without migration background. Z Gerontol Geriatr. 2018. doi: 10.1007/s00391-018-1414-8.
6. Wagner A, Rieger MA, Manser T, Sturm H, Hardt J, Martus P, et al. Healthcare professionals' perspectives on working conditions, leadership, and safety climate: a cross-sectional study. BMC Health Serv Res. 2019; 19: 53. doi: 10.1186/s12913-018-3862-7.
7. Goetz K, Berger S, Gavartina A, Zaroti S, Szecsenyi J. How psychosocial factors affect well-being of practice assistants at work in general medical care?--a questionnaire survey. BMC Fam Pract. 2015; 16: 166. doi: 10.1186/s12875-015-0366-y.
8. Nolting H-D, Berger J, Niemann D, Genz HO, Kordt M. BGW-DAK Stress-Monitoring 2001. Überblick über die Ergebnisse einer BGW-DAK-Studie zum Zusammenhang von Arbeitsbedingungen und Stressbelastung in ausgewählten Berufen; 2001. Available: http://people.f3.htw-berlin.de/Professoren/Pruemper/instrumente/KFZA-BGW-DAK-StressMonitoring_UEBERBLICK.pdf. Accessed 20 March 2019.
9. Paquet M, Courcy F, Lavoie-Tremblay M, Gagnon S, Maillet S. Psychosocial work environment and prediction of quality of care indicators in one Canadian health center. Worldviews Evid Based Nurs. 2013; 10: 82–94. doi: 10.1111/j.1741-6787.2012.00250.x.
10. Theorell T, Hammarstrom A, Aronsson G, Traskman Bendz L, Grape T, Hogstedt C, et al. A systematic review including meta-analysis of work environment and depressive symptoms. BMC Public Health. 2015. doi: 10.1186/s12889-015-1954-4.
11. Bundesanstalt für Arbeitsschutz und Arbeitsmedizin. Volkswirtschaftliche Kosten durch Arbeitsunfähigkeit 2017; 2019. Available: https://www.baua.de/DE/Themen/Arbeitswelt-und-Arbeitsschutz-im-Wandel/Arbeitsweltberichterstattung/Kosten-der-AU/pdf/Kosten-2017.pdf?__blob=publicationFile&v=4. Accessed 20 March 2019.
12. Bundesanstalt für Arbeitsschutz und Arbeitsmedizin. Psychological strain. Available: https://www.baua.de/DE/Themen/Arbeitsgestaltung-im-Betrieb/Psychische-Belastung/_functions/BereichsPublikationssuche_Formular.html?nn=8580646. Accessed 11 March 2019.
13. Lemyre L, Tessier R. Measuring psychological stress. Concept, model, and measurement instrument in primary care research. Can Fam Physician. 2003; 49: 1159-60, 1166-8.
14. Rohmert W, Rutenfranz J. Arbeitswissenschaftliche Beurteilung der Belastung und Beanspruchung an unterschiedlichen industriellen Arbeitsplätzen: Der Bundesminister für Arbeit und Sozialordnung; 1975.
15. Rau R, Henkel D. Zusammenhang von Arbeitsbelastungen und psychischen Erkrankungen. Der Nervenarzt. 2013; 84: 791–798. doi: 10.1007/s00115-013-3743-6.
16. Rau R, Buyken D. Der aktuelle Kenntnisstand über Erkrankungsrisiken durch psychische Arbeitsbelastungen. Zeitschrift für Arbeits- und Organisationspsychologie A&O. 2015; 59: 113–129. doi: 10.1026/0932-4089/a000186.
17. Gesetz über die Durchführung von Maßnahmen des Arbeitsschutzes zur Verbesserung der Sicherheit und des Gesundheitsschutzes der Beschäftigten bei der Arbeit (Arbeitsschutzgesetz - ArbSchG); 2015.
18. Weigl M, Herbig B, Bahemann A, Böckelmann I, Darius S, Jurkschat R, et al. Recommendations on developing and carrying out psychosocial risk evaluations at the workplace. ASU - Arbeitsmed Sozialmed Umweltmed. 2015: 660–665.

19. Hahnzog S. Gesund und glücklich arbeiten – Gefährdungsbeurteilung psychischer Arbeitsbelastung. In: Pfannstiel MA, Mehlich H, editors. BGM – Ein Erfolgsfaktor für Unternehmen: Lösungen, Beispiele, Handlungsanleitungen. Wiesbaden: Springer Fachmedien Wiesbaden; 2018. pp. 681–698.
20. Richter G. Toolbox Version 1.2. - Instrumente zur Erfassung psychischer Belastungen.; 2010. Available: https://www.baua.de/DE/Angebote/Publikationen/Berichte/F1965.html. Accessed 2 April 2019.
21. Viehmann A, Kersting C, Thielmann A, Weltermann B. Prevalence of chronic stress in general practitioners and practice assistants: Personal, practice and regional characteristics. PLoS ONE. 2017; 12: e0176658. doi: 10.1371/journal.pone.0176658.
22. Dreher A, Theune M, Kersting C, Geiser F, Weltermann B. Prevalence of burnout among German general practitioners: Comparison of physicians working in solo and group practices. PLoS ONE. 2019; 14: e0211223. doi: 10.1371/journal.pone.0211223.
23. Viehmann A, Thielmann A, Gesenhues S, Weltermann B. Do Academic Family Practices Reflect Routine Primary Care. Repräsentieren akademische Hausarztpraxen die hausärztliche Regelversorgung. Eine methodische Annäherung. Z. Allg Med. 2014: 354–360.
24. Elm E von, Altman DG, Egger M, Pocock SJ, Gotzsche PC, Vandenbroucke JP. The Strengthening the Reporting of Observational Studies in Epidemiology (STROBE) statement: guidelines for reporting observational studies. J Clin Epidemiol. 2008; 61: 344–349. doi: 10.1016/j.jclinepi.2007.11.008.
25. Prümper J, Hartmannsgruber K, Frese M. KFZA. Kurz-Fragebogen zur Arbeitsanalyse. European Economic Review - EUR ECON REV. 1995; 39.
26. Prümper J. Von der KFZA-Grobanalyse zur IPLV-Feinanalyse Eine Methode zur Maßnahmenentwicklung in der Evaluierung psychischer Belastung. personal manager. 2015; 2.
27. Richter G. Toolbox Version 1.2. Instrumente zur Erfassung psychischer Belastungen ; Forschung Projekt F 1965. 2nd ed. Dortmund: Bundesanstalt für Arbeitsschutz und Arbeitsmedizin; 2011.
28. Allgemeine Unfallversicherungsanstalt (AUVA). Kurzfragebogen zur Arbeitsanalyse. Available: https://fragebogen-arbeitsanalyse.at/login. Accessed 2 April 2019.
29. Leittretter S, editor. Arbeit in Krankenhäusern human gestalten. Arbeitshilfe für die Praxis von Betriebsräten, betrieblichen Arbeitsschutzexperten und Beschäftigten in Krankenhäusern. Düsseldorf: Hans-Böckler-Stiftung; 2008.
30. Orgambidez-Ramos A, Almeida H de. Work engagement, social support, and job satisfaction in Portuguese nursing staff: A winning combination. Appl Nurs Res. 2017; 36: 37–41. doi: 10.1016/j.apnr.2017.05.012.
31. Szecsenyi J, Goetz K, Campbell S, Broge B, Reuschenbach B, Wensing M. Is the job satisfaction of primary care team members associated with patient satisfaction. BMJ Quality & Safety. 2011; 20: 508. doi: 10.1136/bmjqs.2009.038166.
32. Schneider A, Weigl M. Associations between psychosocial work factors and provider mental well-being in emergency departments: A systematic review. PLoS ONE. 2018. doi: 10.1371/journal.pone.0197375.
33. Halter M, Boiko O, Pelone F, Beighton C, Harris R, Gale J, et al. The determinants and consequences of adult nursing staff turnover: a systematic review of systematic reviews. BMC Health Serv Res. 2017. doi: 10.1186/s12913-017-2707-0.

Figure

Click here to access/download;Figure;Fig1.tif

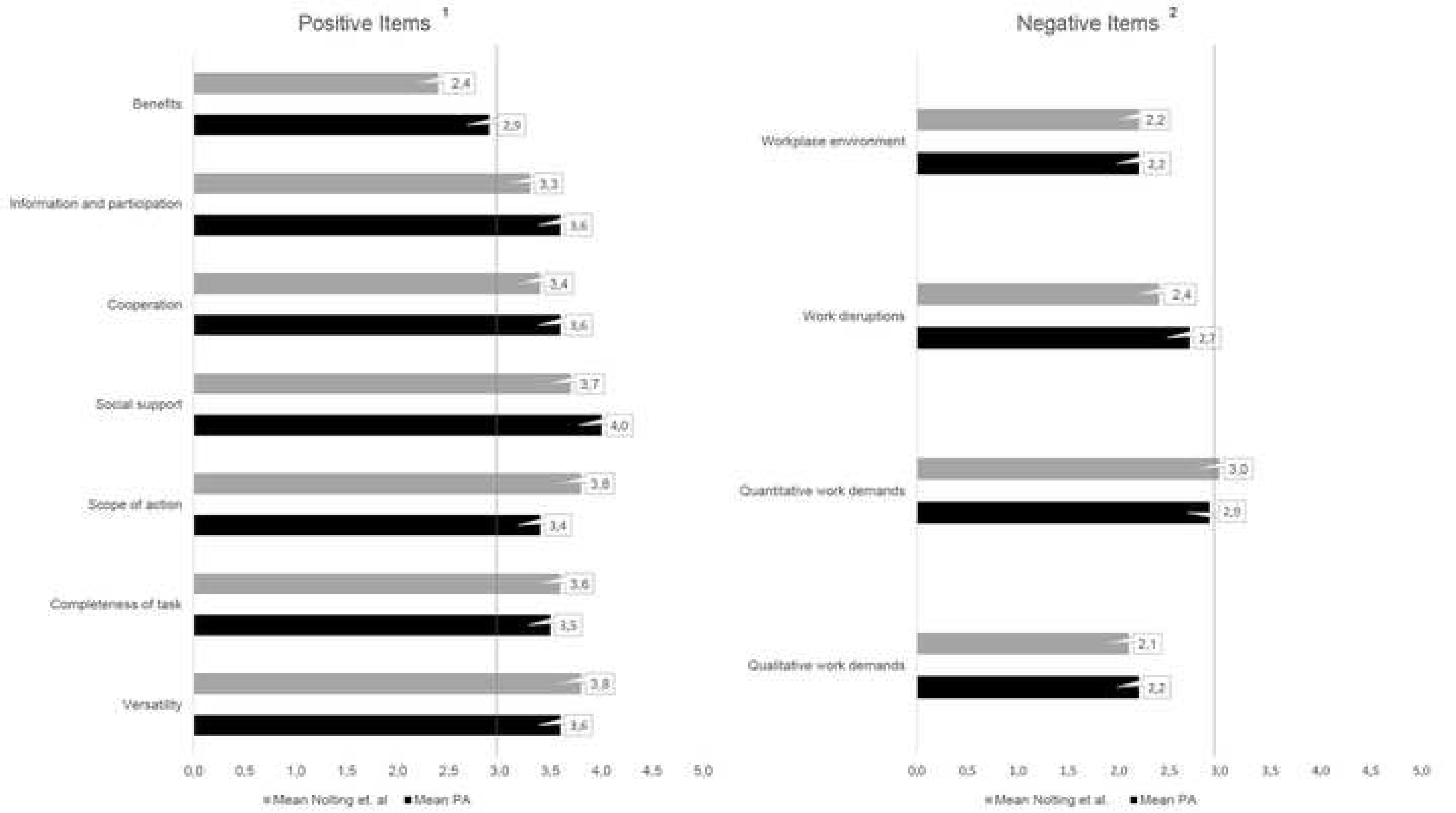

Practice assistants´ perceived mental work-load: Aa cross-sectional study with 550 -German participants addressing work content, stressors, resources, and organizational structure

Jan Hoffmann[1, #a*], Christine Kersting[2, #b], Birgitta Weltermann[1]

[1] Institute of General Practice and Family Medicine, University Hospital of Bonn, 53127 Bonn, Germany

[2] Institute for General Medicine, University Hospital Essen, University of Duisburg-Essen, 45122 Essen, Germany

[#a] – Current Address: Institute of Medical Sociology, Health Services ReserachResearch and Rehabilitation Science, University of Cologne, 50933 Cologne, Germany

[#b] – Current Address: Chair for Innovation and Collaboration in Ambulatory Health Care, Faculty of Health/School of Medicine, Witten/Herdecke University, 58448 Witten, Germany

* Corresponding author

E-mail: jan.hoffmann@uk-koeln.dejan.hoffmann@uk-koeln.de (JH)

Style Definition: Heading 1: Font: +Body (Calibri), Bold, Font color: Text 1

Style Definition: Heading 2: Font: Bold

Formatted: English (United States)

Formatted: English (United States)

Formatted: English (United States)

Formatted: English (United States)

Formatted: English (United States)

Formatted: English (United States)

Formatted: German (Germany)

Formatted: Default Paragraph Font, German

Formatted: Default Paragraph Font, German

Formatted: German (Germany)

Abstract

Formatted: Heading 1, Left

Introduction: Practice assistants~~ (PAs)~~ represent a highly relevant occupational group in Germany and one of the most popular training professions in Germany. ~~Yet~~Despite this, most research in the health care sector has focused on secondary care settings, but has not addressed practice assistants~~PAs~~ in primary care. Knowledge ~~Especially little is known regarding~~ about practice assistants'~~PAs~~ work~~-~~place -related stressors and resources is particularly scarce. This cross-sectional study addresses the mental workload of practice assistants~~,~~ ~~PAs~~ working in primary care practices.

Methods: ~~This cross-sectional study invited p~~Practice assistant~~PA~~s from a network of 185 ~~-~~German primary care practices were invited to participate in this cross-sectional study. The standardized `S~~s~~hort Q~~q~~uestionnaire for W~~w~~orkplace ~~analysis'~~ Analysis'~~'~~ (German: Kurzfragebogen zur Arbeitsanalyse~~, KFZA)~~ ~~(KFZA)~~ was used to assess practice assistants´~~PAs'~~ mental workload. It ~~addressed~~ addresses eleven ~~KFZA~~ workplace factors in 26 items: versatility, completeness of task, scope of action, social support, cooperation, qualitative work demands, quantitative work demands, work disruptions, workplace environment, information and participation, and benefits. ~~Also~~S~~, s~~ocio~~-~~demographic and work characteristics were also obtained~~requested~~. A d~~D~~escriptive analysis was performed for sociodemographic data and ~~KFZA~~ `"~~s~~Short ~~q~~Questionnaire for ~~w~~Workplace ~~a~~Analysis´" factors. The one-sided t-test and Cohen´s d were calculated for a comparison with ~~available~~ data from 23 ~~-~~professional groups (n=8,121).

Results: A total of 550 ~~PAs~~ practice assistants from 130 ~~-~~practices participated. The majority of ~~PAs~~ practice assistants was female (98.5%) and worked full-time (64.5%) in group practices (50.2%). ~~In comparison~~ Compared to the other professional groups, ~~PAs~~ practice assistants reported higher values for the factor social support (4.0 versus~~.~~ 3.7 [d 0.44; p<0.001]), information and participation (3.6 versus~~.~~ 3.3 [d 0.38; p<0.001] as well as work disruptions (2.7 vs. 2.4 [d 0.42; p<0.001]), while ~~PAs~~ practice assistants showed lower values regarding scope of action (3.4 versus~~s.~~ 3.8 [d 0.43; p<0.001]).

Conclusions: Our study identified social support and participation within primary care practices as ~~a~~ protective factors for mental workload, while work disruptions and scope of action were perceived as stressors.

Keywords: practice assistants, primary care, mental workload, psychosocial risk assessment, workplace characteristics

Introduction

Practice assistants (PrAs) represent the largest group of employees in the German ~~ambulatory~~ outpatient health care sector [1] and the second most popular training profession among German wome~~a~~n [2]. However, little is known about how PrAs perceive their work~~ing~~ conditions. More specifically, ~~there is a lack of~~ data on the relationship between work and psychological stress in PrAs are lacking. While ~~a number of studies exist for~~ psychosocial assessment studies of health personnel in secondary care have been performed [3–6], only few ~~studies~~ have addressed this issue in PrAs in German primary care [1,7,8]. Therefore, it is important to further investigate PrAs' ~~generate further evidence for PAs'~~ perceived level of psychological stress, ~~. This is relevant~~ as psychological strain may not only threaten PrAs' health with potentially tremendous economic costs, but may also impair ~~high~~ high-quality patient care [9].

In recent years, increasing attention has been devoted to employees' mental health~~In recent years, a growing interest has been devoted to employees' mental health of employees~~. A systematic review by Theorell ~~and colleagues~~ et al. ~~has~~ highlighted~~showned~~ that ~~evidence for the impact of~~ job strain has an impact on the development of depressive symptoms [10]. Also, the socio-economic implications are increasingly evident: ~~only~~ preceded only by musculo~~muscle~~ skeletal diseases, mental health conditions ~~place~~ rank second with 16.7% of all sick leaves among German employees [11]~~(11)~~ and caused a damage of 21.7 billion Euros ~~loss of~~ gross added ~~gross~~ value in 2017 [11].

Formatted: Heading 1, Left

Formatted: Font: Not Bold

Commented [TR1]: Dies ist ein wenig unglücklich ausgedrückt, da der Fokus meines Erachtens weniger darauf liegen sollte, neue Hinweise bzw. Beweise zu erbringen, sondern das vorhandene Wissen aufzustocken (also allgemein ist die Formulierung "generate evidence" etwas unschön). Ich würde es umformulieren, z.B. "... it is important to further investigate PAs' perceived ...".

Commented [TR2]: Man kann diesem Thema vielleicht mehr Zeit widmen, aber nicht Interesse - hier wäre attention besser. (Zum Beispiel: In recent years, increasing attention has been devoted to employees's mental health.")

The load stress modelstress-strain model developed by Rohmert and Rutenfranz in 1975 differentiates between the terms ‘psychological stress’ as defined above and ‘psychological strain’. The term ‘Ppsychological stress’ describes all external factors that influence one’s psychological well-being. When connecting referring to psychological stress to in a work environment, the term `mental work load´ refers to employees´ expositions exposure to individual work demands and the environment at work [12]. However, tThe term however does not necessarily have imply a negative phenomenon connotation [13]. ‘Psychological strain’ can be understood as the an individual´s immediate response to psychological stress. Thus, the same amount level of psychological stress may elicit lead to a different amount level of psychological strain depending on an employee´s coping strategy and constitution [14]. A well-balanced amount of psychological strain can lead to a healthy and productive workflow [12], while an extreme form level of psychological strain may threaten employees’ health. Studies have shown a negative association between high amounts levels of psychological strain and mental illness [15,16].

Since 2014, the German Safety and Health at Work Act (ArbSchG) law legislation (German Safety and Health at Work Act) obligates employers to perform a general risk assessment of their employees’ working conditions [17]. Part of this risk assessment is the assessment of Assessing the mental workload (a so-called `psychosocial risk assessment´) is part of this risk assessment(so called `psychosocial risk assessment´). Based on such assessmentsthe results, employers must take need to perform counter-measures if as necessary to enhance their employees’ health [18]. Due to differences in work demands, work hazards, and work environments across professions there is no gold standard that defines what on what instrument to should be used for the psychosocial risk assessment. While different instruments exist [19], the so-called KFZA Kurzfragebogen zur Arbeitsanalyse (KFZA; English: sShort qQuestionnaire for wWorkplace aAnalysis), (a short questionnaire addressing perceived workload,) is widely used across professions [20]. with dData from over more than 8,000 000 participants from 23 23 professionals are available [8].

Commented [TR3]: German Occupational Safety and Health Act?

The aims of this cross-sectional study are threefold: i) to assess the mental workload of PrAs working in German primary care practices, ii) to identify resources and stressors, and iii) to compare results with aggregated data from 23 23 different professions.

Material and Methods

Study design and recruitment of participants

The psychosocial assessment of PrAs reported in this paper was obtained as part of a larger cross-sectional study investigating multiple aspects of stress in primary care practices. Details of the study are reported elsewhere [21,22]. Briefly, general practitioners (GPs) and PrAs of the 185 –general medicine practices of the practice network of the Institute for General Medicine, University Hospital Essen, Essen, Germany, were asked to participate in the study. The practices were located in urban and rural regions of North-Rhine-Westphalia (Western Germany) with an average distance of 30 km (range: 2±180 km) to the Iinstitute. In a prior study, it was shown that the practices affiliated with the network are representative for German primary care practices [23]. Practices had been invited by mail and contacted by phone for further recruitment. Those refusing to participate had beenwere asked to answer a short questionnaire on practice characteristics and to provide reasons for non-participation. Data were collected between April and September 2014 during on-site visits. Within each practice, all GPs (practice owners and employed physicians) and PrAs including medical secretaries and PrA trainees were eligible for participation and received the study documents. The study documents comprised a study information sheet, an informed consent form to be completed by all participants, and a set of questionnaires which included sociodemographic questions and the Sshort Qquestionnaire for workplace Workplace analysis Analysis (KFZA) analyzed in this paper. To enassure data protection, participants were asked to seal the completed questionnaire in an envelope. As an incentive, practice teams received a department store chain voucher of 5 –euros per person, irrespective of the

Formatted: Heading 1, Left

Formatted: Font: Not Bold

participation of ~~single~~ individual team members. In addition, the dataset contained information about the practices´ location ~~that was received~~ from the practice network´s database and matched with public regional data for the population size ~~on a~~ in 2012 ~~level~~ (www.it.nrw.de). This paper follows the STROBE recommendations for reporting cross-sectional studies ~~This paper was informed by the STROBE Statement for reporting cross-sectional studies~~ [24].

Ethical approval had been obtained from the Ethics Committee of the Medical Faculty of the University of Duisburg-Essen (reference number: 13-5536-BO, date of approval: 24/11/2014). All participants received written information and signed informed consent forms.

Study instrument to assess mental workload

The ~~short questionnaire for workplace analysis (German: Kurzfragebogen zur Arbeitsanalyse (~~KFZA~~))~~ was developed by Prümper et al.~~and colleagues~~ in 1995 and is ~~serves~~ as a widely ~~well~~ accepted screening tool for psychological stress at the workplace [25]. The questionnaire is a standardized instrument with closed questions. It is ~~filled~~ completed by the employees themselves and thus ~~represents~~ provides a subjective view of each individual's perception of the work environment. According to DIN EN ISO 10075 "E~~e~~rgonomic principles related to mental workload", the instrument is categorized as a "precision level 2 process for overview purposes" [26]. The instrument is listed in the toolbox for "Instruments for recording mental loads" of the Federal Institute for Occupational Safety and Health and covers multiple aspects of the work environment [27]. It ~~covers~~ includes four dimensions: *work content, resources, stressors, and organizational culture.* Dimensions consist of 11 ~~-~~factors which are derived from 26 single items with answer options on a Likert s~~S~~cale ranging from 1 (does not apply at all) to 5 (is completely true). ~~The~~ ~~*dimension*~~ *W~~w~~ork content* ~~dimension~~ contains two factors (versatility, completeness of task) and five single items (learning new skills, use of knowledge, skills and ability, variety of tasks, visibility of task accomplishment, completeness of product). ~~*The dimension*~~ *R~~r~~esources* ~~dimension~~ contains three factors (scope of action, social support, cooperation) and nine single items (influence on sequence of activities, influence on work content,

Commented [TR4]: Was genau ist hiermit gemeint?

Commented [JH5R4]: Hier soll stehen, dass das Paper den STROBE guidelines aus dem STROBE Statement gefolgt ist. Vielleicht so: „This paper follows the STROBE recommendations for reporting cross-sectional studies."

Formatted: Font: Not Bold

Formatted: Space After: 0 pt, Don't adjust space between Latin and Asian text, Don't adjust space between Asian text and numbers

Formatted: Heading 2, Left

Formatted: Font: Italic

Formatted: Font: Italic

Formatted: Font: Italic

Formatted: Font: Italic

influence on work~~-~~load and procedures, social support by co-workers, social support by supervisors, social cohesion within the department, necessity of cooperation, opportunity for social exchange with co-workers, feedback from supervisors and co-workers). ~~*The dimension S*~~*stressors* ~~dimension~~ contains four factors (qualitative work demands, quantitative work demands, work disruptions, workplace environment) and eight single items (excessive complexity of tasks, excessive demands on concentration, frequent work under time pressure, too much work to do, lack of information, work materials or equipment, interruptions of workflow, unfavorable physicochemical conditions, insufficient work~~-~~space and equipment). ~~Lastly , *the dimension O*~~*organizational culture* ~~dimension~~ contains two factors (information and participation, benefits) and four single items (information about organizational developments, consideration of employee input, continuous education, opportunities for advancement). The dimensions job content, resources, and organizational culture represent positive aspects, and high ~~values~~ scores are considered ~~beneficial~~positive. High ~~values~~ scores in the ~~dimension~~ stressors dimension are considered negative work aspects ~~of work~~.

Formatted: Font: Italic

Formatted: Font: Italic

Given the time~~-~~ constraints in primary care practices, the KFZA was deemed ~~chosen as a~~ suitable ~~tool~~ as it takes only 10 minutes ~~time~~ to complete. Also, data from more than 8,000 participants from 23 other professional groups are available for comparison [25]. The questionnaire can be applied throughout all professions and workspaces and is ~~freely~~ readily available for academic use [28].

Comparative data from 23 professional groups

Formatted: Heading 2, Left

In 2000, the Employers' Liability Insurance Association for Medical Services and Welfare Work (BGW) in cooperation with the German Employees' Health Insurance (DAK) conducted a cross-sectional study to measure stress at work [8]. A purposive sample of 27,584 employees from 23 professional groups was selected from the BGW and DAK register: physicians, assistant pharmacists, pharmacists, office workers, teacher, hairdressers, pest controllers, alternative practitioners, unskilled laborers, kindergarten teachers, chefs, nurses, masseurs, medical laboratory technicians, porters, facility

cleaners, social workers, PrAs~~practice assistants~~, veterinarians, care workers for persons at risk, employees of dialysis centers, and employees of workshops for the disabled. A total of 8,121 ~~-~~employees ~~had~~ participated in the~~at~~ study in the context of a project called `Prevention of work-related health hazards'. The KFZA ~~had been~~was used within the scope ~~as part~~ of the~~at~~ study. We performed two comparative analyses using published data of the~~at~~ survey: first, we compared KFZA results from the~~at~~ study ~~for~~ of the 23 ~~-~~professional groups with results from our population. Second, we compared the results for the subpopulation of PrAs from the~~at~~ study with results from our population. The latter comparison is particularly interesting, as it provides a longitudinal approach (data from 2000 and 2014) in a situation where the vocational training was meanwhile been revised ~~meanwhile~~ and PrAs in Germany are professionalizing.

Data analysis

Formatted: Heading 2, Left

The a~~A~~nalysis was performed using IBM SPSS Statistics for Windows, Version 25 (Armonk, NY: IBM Corp.). Data of all PrAs were analyzed. Missing data are reported for all items. Non-plausible values were recoded as missing values.

Socio~~-~~demographic and work-related characteristics were analyzed descriptively. The mean, standard deviation (SD), median, and range are reported f~~F~~or metric socio~~-~~demographic and work variables ~~mean, standard deviation (SD), median, and range are reported~~. The ~~P~~practices' ~~areas´~~ population size was categorized into rural, small, medium-sized, and big cities following categorization schemes of the Federal Institute for Research on Building, Urban Affairs and Spatial Development (rural ≤ 4~~.~~,999 ~~-~~inhabitants, small city 5000~~ – ~~-19~~.~~,999, medium-sized city 20~~.~~,000~~ – ~~-99~~.~~,9999, big city ≥ 100~~.~~,000).

Following Prümper et al.~~and colleagues~~, the results of the ~~evaluation of the~~ KFZA ~~was performed~~ were evaluated by computing mean values on a factor level [25,29]: A~~a~~s a first overview, positive items <3 and negative items >3 ~~respectively~~ are interpreted as high ~~amounts~~ levels of psychological stress and

indicate ~~the~~ a need for more detailed analyses. In addition, the comparison with data from other professional groups or from the same professional group provides information on how to set a benchmark against other results [29]. Differences between the means of our population and the comparative population were analyzed using a ~~one~~ one-sided t-test (95% significance level; 0.05 = alpha). Additionally, Cohen´s d was calculated to estimate the effect size. 95% confidence intervals (CI) were calculated for factors of the 2014 PrA population.

Results

Formatted: Heading 1, Left

Formatted: Font: Not Bold

Study characteristics

550 ~~-~~PrAs ~~had~~ participated in the study (response rate 70.3%; n=130 practices). There were ~~4~~ four implausible values that were recoded as missing values. The socio~~-~~demographic characteristics of the participants are presented in T~~t~~able ~~-~~1. PrAs had a mean age of 37.97 years (SD: 12.63)~~ years~~, with 98.55% of PrAs being female. The majority of PrAs was married (50.364%), worked full~~-~~time (64.55%) ~~in an open-term employment~~on a permanent contract (84.55%) with a median work experience of ~~18~~ 18 years (range~~, ranging from~~: 0-49 years). Most (61.45%) ~~of~~ PrAs worked 20~~ – ~~~~39~~ 39 hours a week, while 24.91% of PrAs worked more than ~~39~~ 39 hours. Most PrAs (90.73%) had ~~finished~~ completed a ~~three year~~three-year vocational training ~~with a degree~~ as “Medizinische Fachangestellte” or “Arzt~~-~~helferin” which combines ~~practice~~ practical training (~~3~~ 3 days per week) and vocational ~~school~~ training (~~2~~ 2 days per week). ~~Ten percent (10.9%)~~Eleven percent had other backgrounds (i.e.: secretary, practice aid, other practice employees~~s~~). Almost all PrAs had completed some sort of additional training:~~.~~ 22.4% of PrAs had completed ~~an~~ additional training as VERAHs (~~106~~ 106 hours of theoretical and ~~94~~ 94 hours of practical training) or EVAs (170 to ~~220~~ 220 hours of theoretical training and 20 to ~~50~~ 50 hours of practical training depending on prior work experience~~s~~) that allows PrAs to perform additional tasks (~~ie~~.eg.: home visits). On average, PrAs worked in practices with 2.96 (SD 2.15)

physicians and 7.73 (SD 7.64) PrAs. Half of ~~all~~ the practices (50.18~~2~~%) were group practices.~~-~~The ~~lowest~~ smallest proportion of PrAs worked in practices with a low patient load ~~number of patients~~ per quarter (5.45%, 501-1000 patients per quarter), while the ~~highest~~ largest proportion of PrAs worked in practices with a high ~~numbers of~~ patient~~s~~ load per quarter (27.27~~3~~%, >3001 ~~-~~patients per quarter). PrA~~a~~s'~~-~~ work setting characteristics are presented in T~~t~~able ~~-~~2.

Table ~~-~~1.~~:~~ Practice assistants´ socio~~-~~demographic and professional training characteristics (n=550)~~-~~.

Variable	Total (n=550)	100%*
**Age (n=550, years)**		
[Mean (SD)]	37.97	(12,.63)
[Median (Minmin-Maxmax)]	38	(16-71)
Missing (n, %)	0	0
**Gender (n, %)**		
Female	542	98.55
Male	4	0.73
Missing	*4*	*0.7*
**Marital status (n, %)**		
Single	218	39.63
Married	277	50.364
Divorced	45	8.182
Widowed	7	1.273
Missing	*3*	*0.5*
**Status of employment (n, %)**		
Full-time	355	64.55
Part-time	179	32.55
Missing	*16*	*2.9*
**Mode of employment (n, %)**		
Fixed-term employment	56	10.182
Open-term employmentPermanent	465	84.55
Missing	*29*	*5.3*
**Working hours per week (n, %)**		
0–-19	65	11.82
20–-39	338	61.45
40–-59	127	23.091

>60	10	1.82
~~*Missing*~~	~~*10*~~	~~*1.8*~~
**Work experience (n=550, years)**		
[Mean (SD)]	18.74	(12,46)
[Median (Min-Max)]	18	(0-49)
~~*Missing (n, %)*~~	~~*10*~~	~~*1.8*~~
**PrA in training (n, %)**		
Yes	49	8.91
No	499	90.73
~~*Missing*~~	~~*2*~~	~~*0.4*~~
**Year of training (n=51, %)**		
First year	16	31.37~~4~~
Second year	19	37.25~~3~~
Third year	12	23.53
~~*Missing*~~	~~*4*~~	~~*7.8*~~
**Vocational training [1] (n, %)**		
Practice assistants	490	89.09~~1~~
Secretary	12	2.18~~2~~
Practice aid[2]	6	1.09~~1~~
Other practice employees[2]	16	2.91
O~~o~~ther~~s~~	75	13.64
~~*Missing*~~	~~*28*~~	~~*5.1*~~
**Additional training (n=137, %)**		
VERAH	14	10.22
EVA	3	2.19~~2~~
VERAH/EVA + other	8	5.84
Other	105	76.64
~~*Missing*~~	~~*7*~~	~~*5.1*~~

[1] multiple answers possible, [2] no vocational training, *numbers do not add up to 100% due to missing values

Table 2~~.~~: Practice assistants' work setting characteristics (n=550)~~.~~

Variable	Total (n=550)	100%
**Type of practice (n, %)**		
Solo practice	147	26.~~,~~73
Group practice	276	50.~~,~~18~~2~~
Others	122	22.1~~,~~8~~2~~
~~*Missing*~~	~~*5*~~	~~*0,9*~~
**Number of patients per quarter (n, %)**		
501-1000	30	5.45
1001-1500	116	21.09~~1~~
1501-2000	100	18.18~~2~~
2001-2500	79	14.36~~4~~
2501-3000	62	11.27~~3~~
>3001	150	27.27~~3~~
~~*Missing*~~	~~*13*~~	~~*2.4*~~
**Location of practice[1] (n, %)**		
Small ~~town~~ city	33	6.00
Medium-sized ~~town~~ city	128	23.27~~3~~
Big city	371	67.45
~~*Missing*~~	~~*18*~~	~~*3.3*~~
**Number of physicians in practice**		
[Mean (SD)]	*2.96*	*(2.15)*
[Median (Min-Max)]	*2*	*(1-10)*
~~*Missing (n, %)*~~	~~*5*~~	~~*0.9*~~
**Number of PrAs in practice**		

Formatted: Font: 11 pt

Formatted: Font: Bold

Formatted: Font: Bold

Formatted: Font: 10 pt (×24)

[Mean (SD)]	*7.73*	*(7.64)*
[Median (Min-Max)]	*5*	*(0-35)*
~~*Missing (n, %)*~~	~~*33*~~	~~*6.0*~~

[1] based on 2012 number of inhabitants, *numbers do not add up to 100% due to missing values

Comparison of practice assistants with other professional groups (comparative data)

Table ~~-~~3 shows the results of the KFZA analysis for PrAs and for the comparative population. For a first overview of only results from our study population, the calculation of mean values for the ~~factor~~ factor-level analysis yielded a critical ~~value~~ score for the factor benefits (2.86 [SD 1.05]). In contrast, social support showed the highest positive factor (4.05 [SD 0.79]).

As illustrated in Fig~~ure~~ ~~-~~1, t~~T~~he comparison of our results with data from Nolting et al. [8] revealed statistically significant differences ($p < 0.05$) for the following factors: versatility (3.6 vs. 3.8), completeness of task (3.5 vs. 3.6), scope of action (3.4 vs. 3.8), social support (4.0 vs. 3.7), cooperation (3.6 vs. 3.4), qualitative work demands (2.2 vs. 2.1), work~~s~~ disruptions (2.7 vs. 2.4), information and participation (3.6 vs. 3.3), and benefits (2.9 vs. 2.4). The two ~~non-significant~~ factors ~~were~~ workplace environment (2.2 vs. 2.2) and quantitative work demands (2.9 vs. 3.0) were found to be non-significant.

Effect size showed the strongest difference for the factors social support (4.0 vs 3.7 [d 0.44]), scope of action (3.4 vs. 3.8 [d 0.43]), and ~~(~~benefits (2.9 vs. 2.4 [d 0.43]). The ~~values~~ scores for social support and benefits were higher in the PrA population than in the comparative group, whereas scope of action yielded lower ~~values~~scores. ~~However, t~~The factor benefits, on the other hand, was critically low in both populations. The difference in work disruptions (2.7 vs. 2.4 [d 0.41]) presented a moderate effect size. The score for ~~re were higher~~ work disruptions was higher in the PrA population compared to the population from Nolting et al. [8].

Formatted: Font: 10 pt

Formatted: Font: 10 pt

Formatted: Font: 10 pt

Formatted: Font: 11 pt

Formatted: Heading 2, Left

Comparison of practice assistants from 2000 and 2014

Formatted: Heading 2, Left

Table ~~-~~4 shows ~~a~~the~~The~~ comparison ~~of~~ between PrAs~~practice assistants~~ in our study population (from 2014) and the comparative study population (from 2000). The comparison yielded statistically significant differences ($p < 0.05$) for the factors completeness of task (3.5 vs. 3.2), social support (4.0 vs. 3.9), cooperation (3.6 vs. 3.5), qualitative work demands (2.2 vs. 2.0), quantitative work demands (2.9 vs. 2.8), work disruptions (2.7 vs. 2.5), workplace environment (2.2 vs. 2.0), information and participation (3.6 vs. 3.5), and benefits (2.9 vs 2.2).

Effect size showed no effect for versatility (d -0.05), scope of action (d -0.01), social support (d -0.19), cooperation (~~d~~ d 0.13), quantitative work demands (~~d~~ d 0.12), as well as information and participation (~~d~~ d 0.16). A s~~S~~mall effect size was shown for completeness of task (~~d~~ d 0.32), qualitative work demands (~~d~~ d 0.25), work disruptions (~~d~~ d 0.29), and workplace environment (~~d~~ d 0.21). The difference in the factor benefits presented a moderate effect size (~~d~~ d 0.62).

Table 3. KFZA results from our study of practice assistants (n=550) in comparison with comparative data from 23 professional groups (n= 8.121).

Work aspects	KFZA factor	Our study Mean Score (PrAs)	95% CI	Comparison: Mean Score (Nolting et al.)	Cohen´s d	P-value **
**Job content[1]**	**Versatility**	**3.6**	3.58 - 3.70	3.8	0.23	< 0.001
	**Completeness of task**	**3.5**	3.41 - 3.57	3.6	0.12	0.0045
**Resources[1]**	**Scope of action**	**3.4**	3.37 - 3.49	3.8	0.43	< 0.001
	**Social support**	**4.0**	3.98 - 4.12	3.7	0.44	< 0.001
	**Cooperation**	**3.6**	3.53 - 3.66	3.4	0.24	< 0.001
**Stressors[2]**	**Qualitative work demands**	**2.2**	2.14 - 2.29	2.1	0.13	0.0025
	**Quantitative work demands**	**2.9**	2.83 - 3.01	3.0	0.07	0.0797
	**Work disruptions**	**2.7**	2.67 - 2.81	2.4	0.41	< 0.001
	**Workplace environment**	**2.2**	2.13 - 2.30	2.2	0.02	0.7109
**Organizational culture[1]**	**Information and participation**	**3.6**	3.57 - 3.73	3.3	0.38	< 0.001
	**Benefits**	**2.9***	2.77 - 2.94	2.4*	0.43	< 0.001

Formatted: Font: Bold

Formatted: Font: Bold

[1] High values scores (>3) are considered positive, [2] high values scores (>3) are considered negative, * critical values ** based on a one sidedone-sided t-test comparing mean values of PrAs and Nolting et al. on a 95% significance level

Figure 1.: KFZA results on a factor level divided into resources and stressors in comparison with comparative data from Nolting et al.

[1] High values scores (>3) are considered positive, [2] high values scores (>3) are considered negative.

Table 4.: KFZA factor factor-level comparison of PrAs from our study (n=550; year 2014) and PrAs from Nolting et al. (n=324; year 2000).

**Work aspects**	**KFZA factor**	**Our study Mean sScore (PrAs)**	95% CI	PrAs' results from 20002 Mean sScore (PrAs; Nolting et al.)	Cohen´s d	P-value
**Job content[1]**	**Versatility**	**3.6**	3.58 - 3.70	3.6	0.05	0.238
	**Completeness of task**	**3.5**	3.41 - 3.57	3.2	0.32	< 0.001
**Resources[1]**	**Scope of action**	**3.4**	3.37 - 3.49	3.4	0.01	0.765
	**Social support**	**4.0**	3.98 -– 4.12	3.9	0.19	< 0.001
	**Cooperation**	**3.6**	3.53 -– 3.66	3.5	0.13	0.006
**Stressors[2]**	**Qualitative work demands**	**2.2**	2.14 -– 2.29	2.0	0.25	< 0.001

Formatted: Font: 11 pt
Formatted: Font: 11 pt
Formatted: Font: 11 pt
Formatted: Font: 11 pt, Bold
Formatted: Font: 11 pt
Formatted: Font: 11 pt, Not Superscript/ Subscript
Formatted: Font: 11 pt
Formatted: Font: Bold
Formatted: Font: Bold
Formatted: Font: Bold
Formatted: Font: Bold
Formatted Table

	**Quantitative work demands**	**2.9**	2.83 -– 3.01	2.8	0.12	0.007
	**Work disruptions**	**2.7**	2.67 -– 2.81	2.5	0.29	< 0.001
	**Workplace environment**	**2.2**	2.13 -– 2.30	2.0	0.21	< 0.001
**Organizational culture**[1]	**Information and participation**	**3.6**	3.57 -– 3.73	3.5	0.16	0.002
	**Benefits**	**2.9***	2.77 – 2.94	2.2*	0.62	< 0.001

[1] High scoresvalues (>3) are considered positive, [2] high values scores (>3) are considered negative, * critical values ** based on a one sidedone-sided t-test comparing mean values of PrAs and Nolting et al. on a 95% significance level

Discussion

Our study identified social support within primary care practices as a resource and a protective factor for mental work~~-~~load among PrAs, while the lack of benefits at work was perceived as a stressor.

When comparing data on PrAs with the aggregated data of other professional groups, we were able to perform a more informative analysis ~~was possible~~ yielding slightly different results. Scope of action and work disruptions showed the largest negative difference and the strongest effect size, whereas social support and benefits showed the largest positive difference and the strongest effect size. Interestingly, when comparing with other professional groups, the factor benefits that was ~~interpreted~~ identified as a stressor in the single evaluation turned out to be a resource ~~when comparing with other professional groups~~. Since ~~values~~ the scores are rather low in both samples, lack of benefits at work might be a general problem, ~~whereas~~ while PrAs might experience more benefits at work ~~compared to~~ than other professional groups. PrAs in general practices tend to be responsible for a wide range of tasks in different workplaces throughout the practices, as they ~~while~~ are~~presenting~~ the first point of contact for patients with unexpected events occurring on a regular basis [1]. This job profile may explain the high ~~values~~ scores for work disruptions. Although PrAs are responsible for a wide range of tasks, GPs remain the decision makers, resulting in ~~leading to~~ a setting-immanent limited scope of action for PrAs.

The c~~C~~omparison between the ~~professional groups of~~ PrA groups from 2000 to 2014 ~~showed~~ revealed significant differences for~~in~~ most factors, but small effect sizes. The factor benefits showed a moderate effect size in favor of the 2014 study population. All factors, positive factors and negative factors alike, were slightly higher in our population of PrAs compared to the 2000 PrA population ~~of PAs~~ from Nolting et al~~. from 2000, positive factors and negative factors alike~~. The increase ~~of~~ in benefits at work and completeness of task from 2000 to 2014 may be explained by ~~a number of~~ the ~~possible~~ further training~~s~~ opportunities ~~for~~ for PrAs that were ~~had been~~ introduced during that time period (i.e., VERAH, EVA). Among other changes, these trainings have enabled PrAs ~~opened up the possibilities for PAs~~ to carry

Formatted: Heading 1, Left

Formatted: Font: Not Bold

out more complex work processes ~~at work~~ autonomously (~~i.e~~e.g.: patient education on diabetes). Additionally, they are rewarded with a better salary. Both may be signs ~~for a~~ of professionalization. ~~PAs i~~In a ~~-~~recent study ~~from~~ by Vu-Eickmann et al., PrAs reported a high patient volume, which in addition to handling many tasks at once may explain the ~~be the reason for~~ high score for work disruptions [1].

Social support is an important resource and can positively influence job satisfaction, as shown in a recent study with Portuguese nursing staff [30]. Job satisfaction was again ~~has been~~ shown to positively correlate with patient satisfaction [31]. A systematic review yielded a similar result ~~connecting~~ linking social support with staff well-being in emergency departments [32]. In contrast, studies have shown that negative work aspect (i.e.: lack of benefits, limited scope of action) cause psychological strain and can lead to a higher turnover rate and depressive symptoms [10,33].

In agreement with three other studies ~~available~~ on ~~the~~ this topic, we showed that PrAs in primary care practices receive high social support and have a rather limited scope of action and still insufficient benefits at work [1,7,8].

Strengths and limitations

It is a strength of our study that it was based on a data set with a large number of participants (550 PrAs). Also, prior analyses had shown that the practice network from which this sample was taken is representative for German primary care practices ~~With 550 participating PAs, our study comprised had a high large number of participants and a high response rate of 70.3%. Additionally, it was shown that the practice network from which this sample was taken is representative for German primary care practices~~ [23]. Each participant received an incentive in the form of a ~~5~~ 5-Euro voucher to avoid a selection bias by ~~only~~ selecting only highly motivated PrAs. As the network is located in a rather densely populated area, our results may over~~-~~represent PrAs working in urban areas. The KFZA proved to be ~~an implementation economic~~a cost-effective screening tool to gain first insights into employees'

Formatted: Heading 2, Left

Commented [JH6]: Vom Reviewer markiert

psychological stressors and resources. To our knowledge this is the first study comparing PrAs’ data from a psychological risk assessment in primary care with a large sample from other professions.

In ~~this~~ our study we were ~~it was~~ only ~~possible~~ able to ~~measure~~ assess the current situation and not the state desired by PrAs, which could have ~~given~~ provided even ~~further~~ more insights. The c~~C~~omparison with data from ~~23~~ 23 professional groups was limited as only aggregated mean results were available without standard deviations. Due to this, we were unable to calculate ~~a calculation of~~ confidence intervals for both populations ~~was not possible~~. A strength of our study is the comparison of the results of the 2000 with the 2014 ~~results~~ study from the same professional group. ~~Yet~~However, the PrA ~~these were two different~~ populations ~~of PAs~~ were not identical, and caution is advised when interpreting the results.

Conclusions

Mental well-being has a tremendous impact on preserving a healthy and productive workforce. Therefore, our ~~it has to be the~~ goal must be to first identify risk factors for mental well-being at work and put them into perspective with other occupations, which we aimed to do in ~~as were the aims of~~ this study. Second, we need to develop measures ~~need to be developed~~ to tackle risk factors for psychological strain at work and enhance protective factors such as social support, scope of action, benefits at work, and cooperation. Last~~ly~~, measures need to be evaluated and implemented in the everyday working life of PrAs.

Formatted: Heading 1, Left

Formatted: Font: Not Bold

Commented [JH7]: Markiert vom Reviewer

List of abbreviations

CI: confidence interval; GP: ~~General~~ general ~~Practitioner~~practitioner; KFZA: Kurzfragebogen zur Arbeitsanalyse (English: ~~short~~ Short ~~questionnaire~~ Questionnaire for ~~workplace~~ Workplace ~~analysis~~Analysis); PrA: ~~Practice~~ practice assistant; SD: ~~Standard~~ standard deviation

Acknowledgements

We thank the Institute for General Medicine, University Hospital Essen, for supporting the conceptualization of the questionnaire, the data collection, and the provision of the data for this analysis.

References

1. Vu-Eickmann P, Loerbroks A. Psychosoziale Arbeitsbedingungen Medizinischer Fachangestellter: Ergebnisse einer qualitativen Studie zu den berufsspezifischen Belastungen, Ressourcen, Präventionsmöglichkeiten und Interventionsbedürfnissen. Z Evid Fortbild Qual Gesundhwes. 2017; 126: 43–51. doi: 10.1016/j.zefq.2017.06.005.
2. Statistisches Bundesamt. Auszubildene. nach Ausbildungsberufen 2017 (TOP 20), Frauen. Available: https://www.destatis.de/DE/ZahlenFakten/GesellschaftStaat/BildungForschungKultur/BeruflicheBildung/Tabellen/AzubiRangliste.html. Accessed 12 March 2019.
3. Freimann T, Merisalu E. Work-related psychosocial risk factors and mental health problems amongst nurses at a university hospital in Estonia: a cross-sectional study. Scand J Public Health. 2015; 43: 447–452. doi: 10.1177/1403494815579477.
4. Kern M, Buia A, Tonus C, Weigel TF, Dittmar R, Hanisch E, et al. Psychological stressors, resources and well-being of surgeons in Germany : A cross-sectional study. Chirurg. 2019. doi: 10.1007/s00104-018-0780-5.
5. Ulusoy N, Wirth T, Lincke H-J, Nienhaus A, Schablon A. Psychosocial burden and strains in geriatric nursing: comparison of nursing personnel with and without migration background. Z Gerontol Geriatr. 2018. doi: 10.1007/s00391-018-1414-8.
6. Wagner A, Rieger MA, Manser T, Sturm H, Hardt J, Martus P, et al. Healthcare professionals' perspectives on working conditions, leadership, and safety climate: a cross-sectional study. BMC Health Serv Res. 2019; 19: 53. doi: 10.1186/s12913-018-3862-7.

Formatted: Font: Not Bold

Formatted: Heading 1, Left, Adjust space between Latin and Asian text, Adjust space between Asian text and numbers

Formatted: Space After: 0 pt, Don't adjust space between Latin and Asian text, Don't adjust space between Asian text and numbers

Formatted: Heading 1 Char, Font: 11 pt, Not Bold, German (Germany)

Field Code Changed

Formatted: German (Germany)

7. Goetz K, Berger S, Gavartina A, Zaroti S, Szecsenyi J. How psychosocial factors affect well-being of practice assistants at work in general medical care?--a questionnaire survey. BMC Fam Pract. 2015; 16: 166. doi: 10.1186/s12875-015-0366-y.
8. Nolting H-D, Berger J, Niemann D, Genz HO, Kordt M. BGW-DAK Stress-Monitoring 2001. Überblick über die Ergebnisse einer BGW-DAK-Studie zum Zusammenhang von Arbeitsbedingungen und Stressbelastung in ausgewählten Berufen; 2001. Available: http://people.f3.htw-berlin.de/Professoren/Pruemper/instrumente/KFZA-BGW-DAK-StressMonitoring_UEBERBLICK.pdf. Accessed 20 March 2019.
9. Paquet M, Courcy F, Lavoie-Tremblay M, Gagnon S, Maillet S. Psychosocial work environment and prediction of quality of care indicators in one Canadian health center. Worldviews Evid Based Nurs. 2013; 10: 82–94. doi: 10.1111/j.1741-6787.2012.00250.x.
10. Theorell T, Hammarstrom A, Aronsson G, Traskman Bendz L, Grape T, Hogstedt C, et al. A systematic review including meta-analysis of work environment and depressive symptoms. BMC Public Health. 2015. doi: 10.1186/s12889-015-1954-4.
11. Bundesanstalt für Arbeitsschutz und Arbeitsmedizin. Volkswirtschaftliche Kosten durch Arbeitsunfähigkeit 2017; 2019. Available: https://www.baua.de/DE/Themen/Arbeitswelt-und-Arbeitsschutz-im-Wandel/Arbeitsweltberichterstattung/Kosten-der-AU/pdf/Kosten-2017.pdf?__blob=publicationFile&v=4. Accessed 20 March 2019.
12. Bundesanstalt für Arbeitsschutz und Arbeitsmedizin. Psychological strain. Available: https://www.baua.de/DE/Themen/Arbeitsgestaltung-im-Betrieb/Psychische-Belastung/_functions/BereichsPublikationssuche_Formular.html?nn=8580646. Accessed 11 March 2019.
13. Lemyre L, Tessier R. Measuring psychological stress. Concept, model, and measurement instrument in primary care research. Can Fam Physician. 2003; 49: 1159-60, 1166-8.
14. Rohmert W, Rutenfranz J. Arbeitswissenschaftliche Beurteilung der Belastung und Beanspruchung an unterschiedlichen industriellen Arbeitsplätzen: Der Bundesminister für Arbeit und Sozialordnung; 1975.
15. Rau R, Henkel D. Zusammenhang von Arbeitsbelastungen und psychischen Erkrankungen. Der Nervenarzt. 2013; 84: 791–798. doi: 10.1007/s00115-013-3743-6.
16. Rau R, Buyken D. Der aktuelle Kenntnisstand über Erkrankungsrisiken durch psychische Arbeitsbelastungen. Zeitschrift für Arbeits- und Organisationspsychologie A&O. 2015; 59: 113–129. doi: 10.1026/0932-4089/a000186.
17. Gesetz über die Durchführung von Maßnahmen des Arbeitsschutzes zur Verbesserung der Sicherheit und des Gesundheitsschutzes der Beschäftigten bei der Arbeit (Arbeitsschutzgesetz - ArbSchG); 2015.
18. Weigl M, Herbig B, Bahemann A, Böckelmann I, Darius S, Jurkschat R, et al. Recommendations on developing and carrying out psychosocial risk evaluations at the workplace. ASU - Arbeitsmed Sozialmed Umweltmed. 2015: 660–665.
19. Hahnzog S. Gesund und glücklich arbeiten – Gefährdungsbeurteilung psychischer Arbeitsbelastung. In: Pfannstiel MA, Mehlich H, editors. BGM – Ein Erfolgsfaktor für Unternehmen: Lösungen, Beispiele, Handlungsanleitungen. Wiesbaden: Springer Fachmedien Wiesbaden; 2018. pp. 681–698.
20. Richter G. Toolbox Version 1.2. - Instrumente zur Erfassung psychischer Belastungen.; 2010. Available: https://www.baua.de/DE/Angebote/Publikationen/Berichte/F1965.html. Accessed 2 April 2019.
21. Viehmann A, Kersting C, Thielmann A, Weltermann B. Prevalence of chronic stress in general practitioners and practice assistants: Personal, practice and regional characteristics. PLoS ONE. 2017; 12: e0176658. doi: 10.1371/journal.pone.0176658.

Formatted: German (Germany)

Formatted: German (Germany)

Formatted: German (Germany)

Formatted: German (Germany)

22. Dreher A, Theune M, Kersting C, Geiser F, Weltermann B. Prevalence of burnout among German general practitioners: Comparison of physicians working in solo and group practices. PLoS ONE. 2019; 14: e0211223. doi: 10.1371/journal.pone.0211223.
23. Viehmann A, Thielmann A, Gesenhues S, Weltermann B. Do Academic Family Practices Reflect Routine Primary Care. Repräsentieren akademische Hausarztpraxen die hausärztliche Regelversorgung. Eine methodische Annäherung. Z. Allg Med. 2014: 354–360.
24. Elm E von, Altman DG, Egger M, Pocock SJ, Gotzsche PC, Vandenbroucke JP. The Strengthening the Reporting of Observational Studies in Epidemiology (STROBE) statement: guidelines for reporting observational studies. J Clin Epidemiol. 2008; 61: 344–349. doi: 10.1016/j.jclinepi.2007.11.008.
25. Prümper J, Hartmannsgruber K, Frese M. KFZA. Kurz-Fragebogen zur Arbeitsanalyse. European Economic Review - EUR ECON REV. 1995; 39.
26. Prümper J. Von der KFZA-Grobanalyse zur IPLV-Feinanalyse Eine Methode zur Maßnahmenentwicklung in der Evaluierung psychischer Belastung. personal manager. 2015; 2.
27. Richter G. Toolbox Version 1.2. Instrumente zur Erfassung psychischer Belastungen ; Forschung Projekt F 1965. 2nd ed. Dortmund: Bundesanstalt für Arbeitsschutz und Arbeitsmedizin; 2011.
28. Allgemeine Unfallversicherungsanstalt (AUVA). Kurzfragebogen zur Arbeitsanalyse. Available: https://fragebogen-arbeitsanalyse.at/login. Accessed 2 April 2019.
29. Leittretter S, editor. Arbeit in Krankenhäusern human gestalten. Arbeitshilfe für die Praxis von Betriebsräten, betrieblichen Arbeitsschutzexperten und Beschäftigten in Krankenhäusern. Düsseldorf: Hans-Böckler-Stiftung; 2008.
30. Orgambidez-Ramos A, Almeida H de. Work engagement, social support, and job satisfaction in Portuguese nursing staff: A winning combination. Appl Nurs Res. 2017; 36: 37–41. doi: 10.1016/j.apnr.2017.05.012.
31. Szecsenyi J, Goetz K, Campbell S, Broge B, Reuschenbach B, Wensing M. Is the job satisfaction of primary care team members associated with patient satisfaction. BMJ Quality & Safety. 2011; 20: 508. doi: 10.1136/bmjqs.2009.038166.
32. Schneider A, Weigl M. Associations between psychosocial work factors and provider mental well-being in emergency departments: A systematic review. PLoS ONE. 2018. doi: 10.1371/journal.pone.0197375.
33. Halter M, Boiko O, Pelone F, Beighton C, Harris R, Gale J, et al. The determinants and consequences of adult nursing staff turnover: a systematic review of systematic reviews. BMC Health Serv Res. 2017. doi: 10.1186/s12913-017-2707-0.

Formatted: German (Germany)

Formatted: German (Germany)

Response to Editor
PONE-D-20-07803
Practice assistants´ perceived mental work load: A cross-sectional study with 550 German participants addressing work content, stressors, resources, and organizational structure

PLOS ONE

Dear Dr. Useche,

We like to thank you and the reviewers for the very helpful advices.
Please find our revision and answers to the open points enclosed.

Best regards,
Jan Hoffmann

Journal Requirements:

When submitting your revision, we need you to address these additional requirements.

1. Please ensure that your manuscript meets PLOS ONE's style requirements, including those for file naming. The PLOS ONE style templates can be found at

https://journals.plos.org/plosone/s/file?id=wjVg/PLOSOne_formatting_sample_main_body.pdf
and

https://journals.plos.org/plosone/s/file?id=ba62/PLOSOne_formatting_sample_title_authors_affiliations.pdf

Answer: Our manuscript now meets all style requirements.

2. We suggest you thoroughly copyedit your manuscript for language usage, spelling, and grammar. If you do not know anyone who can help you do this, you may wish to consider employing a professional scientific editing service.

Whilst you may use any professional scientific editing service of your choice, PLOS has partnered with both American Journal Experts (AJE) and Editage to provide discounted services to PLOS authors. Both organizations have experience helping authors meet PLOS guidelines and can provide language editing, translation, manuscript formatting, and figure formatting to ensure your manuscript meets our submission guidelines. To take advantage of our partnership with AJE, visit the AJE website (http://learn.aje.com/plos/) for a 15% discount off AJE services. To take advantage of our partnership with Editage, visit the Editage website (www.editage.com) and enter referral code PLOSEDIT for a 15% discount off Editage services. If the PLOS editorial team finds any language issues in text that either AJE or Editage has edited, the service provider will re-edit the text for free.

Upon resubmission, please provide the following:

a) The name of the colleague or the details of the professional service that edited your manuscript

b) A copy of your manuscript showing your changes by either highlighting them or using track changes (uploaded as a *supporting information* file)

c) A clean copy of the edited manuscript (uploaded as the new *manuscript* file)

Answer: The manuscript was proofread by a professional medical translator (Sarah Chalmers; https://www.medi-translate.com/)

3. We note that you have indicated that data from this study are available upon request. PLOS only allows data to be available upon request if there are legal or ethical restrictions on sharing data publicly. For information on unacceptable data access restrictions, please see http://journals.plos.org/plosone/s/data-availability#loc-unacceptable-data-access-restrictions.

In your revised cover letter, please address the following prompts:

a) If there are ethical or legal restrictions on sharing a de-identified data set, please explain them in detail (e.g., data contain potentially identifying or sensitive patient information) and who has imposed them (e.g., an ethics committee). Please also provide contact information for a data access committee, ethics committee, or other institutional body to which data requests may be sent.

b) If there are no restrictions, please upload the minimal anonymized data set necessary to replicate your study findings as either Supporting Information files or to a stable, public repository and provide us with the relevant URLs, DOIs, or accession numbers. Please see http://www.bmj.com/content/340/bmj.c181.long for guidelines on how to de-identify and prepare clinical data for publication. For a list of acceptable repositories, please see http://journals.plos.org/plosone/s/data-availability#loc-recommended-repositories.

We will update your Data Availability statement on your behalf to reflect the information you provide.

Answer: The data cannot be shared publicly because of ethical restrictions and data protection issues as our dataset includes potentially identifying information.

4. Please ensure that you refer to Figure 1 in your text as, if accepted, production will need this reference to link the reader to the figure.

Answer: A reference to Figure 1 is now included (line 208).

"As illustrated in Fig 1, the comparison of our results with data from Nolting et al. [8] revealed statistically significant differences ($p < 0.05$) for the following factors: versatility (3.6

vs. 3.8), completeness of task (3.5 vs. 3.6), scope of action (3.4 vs. 3.8), social support (4.0 vs. 3.7), cooperation (3.6 vs. 3.4), qualitative work demands (2.2 vs. 2.1), works disruptions (2.7 vs. 2.4), information and participation (3.6 vs. 3.3), and benefits (2.9 vs. 2.4).”

5. We note you have included a table to which you do not refer in the text of your manuscript. Please ensure that you refer to Table 4 in your text; if accepted, production will need this reference to link the reader to the Table.

Answer: A reference to Table 4 is now included (line 221).

“Table 4 shows a comparison of PrAs in our study population (from 2014) and the comparative study population (from 2000).”

[Note: HTML markup is below. Please do not edit.]

Reviewers' comments:

Reviewer's Responses to Questions

Comments to the Author

1. Is the manuscript technically sound, and do the data support the conclusions?

The manuscript must describe a technically sound piece of scientific research with data that supports the conclusions. Experiments must have been conducted rigorously, with appropriate controls, replication, and sample sizes. The conclusions must be drawn appropriately based on the data presented.

Reviewer #1: Yes

---

2. Has the statistical analysis been performed appropriately and rigorously?

Reviewer #1: Yes

---

3. Have the authors made all data underlying the findings in their manuscript fully available?

The PLOS Data policy requires authors to make all data underlying the findings described in their manuscript fully available without restriction, with rare exception (please refer to the Data Availability Statement in the manuscript PDF file). The data should be provided as part of the manuscript or its supporting information, or deposited to a public repository. For example, in addition to summary statistics, the data points behind means, medians and variance measures

should be available. If there are restrictions on publicly sharing data—e.g. participant privacy or use of data from a third party—those must be specified.

Reviewer #1: No

Answer: The data cannot be shared publicly because of ethical restrictions and data protection issues as our dataset includes potentially identifying information.

---

4. Is the manuscript presented in an intelligible fashion and written in standard English?

PLOS ONE does not copyedit accepted manuscripts, so the language in submitted articles must be clear, correct, and unambiguous. Any typographical or grammatical errors should be corrected at revision, so please note any specific errors here.

Reviewer #1: No

Answer: The manuscript was proofread by a certified medical translator.

---

5. Review Comments to the Author

Please use the space provided to explain your answers to the questions above. You may also include additional comments for the author, including concerns about dual publication, research ethics, or publication ethics. (Please upload your review as an attachment if it exceeds 20,000 characters)

Reviewer #1:

1. The manuscript is full of typography errors; punctuations.
Answer: The manuscript was proofread by a professional medical translator.

2. Language is main problem
Answer: The manuscript was proofread by a professional medical translator.

3. Not consistent throughout the document
Answer: The manuscript was proofread by a professional medical translator.

4. Don’t use abbreviation in the abstract part
Answer: This was corrected.

5. In the background part there are incomplete sentences
Answer: This was corrected.

6. In tables the decimal places should be consistent
Answer: This was corrected in Tables 1, 2,3 and 4.

7. In the table reporting missing value is not necessary
Answer: This was corrected in Tables 1 and 2.

---

6. PLOS authors have the option to publish the peer review history of their article (what does this mean?). If published, this will include your full peer review and any attached files.

If you choose "no", your identity will remain anonymous but your review may still be made public.

Do you want your identity to be public for this peer review? For information about this choice, including consent withdrawal, please see our Privacy Policy.

Reviewer #1: No

Answer to reviewer comment concerning response rate

Reviewer comment: "Statistically how it can be generalized with around 30% non-response rate?

Answer: The argument in our sentence was incorrect. The total study had a response rate of 70% of practices. Within the practices, nearly all physicians and practice assistants participated indicating a high interest in the topic.

The text was revised to: It is a strength of our study that it was based on a data set with a large number of participants (550 PrAs). Also, prior analyses had shown that the practice network from which this sample was taken is representative for German primary care practices.
