# Supplementary material for: Practice assistants´ perceived mental workload: A cross-sectional study with 550 German participants addressing work content, stressors, resources, and organizational structure
Source: PLoS One. 2020 Oct 1;15(10):e0240052. doi: 10.1371/journal.pone.0240052 (PMC7529273; doi:10.1371/journal.pone.0240052)
Supplement: Attachment — Submitted filename: Response to Reviewers.docx [file pone.0240052.s001.docx]

**Response to Editor**

PONE-D-20-07803
Practice assistants´ perceived mental work load: A cross-sectional study with 550 German participants addressing work content, stressors, resources, and organizational structure

PLOS ONE

*Dear Dr. Useche,*

*We like to thank you and the reviewers for the very helpful advices.*

*Please find our revision and answers to the open points enclosed.*

*Best regards,*

*Jan Hoffmann*

Journal Requirements:

When submitting your revision, we need you to address these additional requirements.

1. Please ensure that your manuscript meets PLOS ONE's style requirements, including those for file naming. The PLOS ONE style templates can be found at

<https://journals.plos.org/plosone/s/file?id=wjVg/PLOSOne_formatting_sample_main_body.pdf> and

<https://journals.plos.org/plosone/s/file?id=ba62/PLOSOne_formatting_sample_title_authors_affiliations.pdf>

Answer: Our manuscript now meets all style requirements.

2.  We suggest you thoroughly copyedit your manuscript for language usage, spelling, and grammar. If you do not know anyone who can help you do this, you may wish to consider employing a professional scientific editing service.  

Whilst you may use any professional scientific editing service of your choice, PLOS has partnered with both American Journal Experts (AJE) and Editage to provide discounted services to PLOS authors. Both organizations have experience helping authors meet PLOS guidelines and can provide language editing, translation, manuscript formatting, and figure formatting to ensure your manuscript meets our submission guidelines. To take advantage of our partnership with AJE, visit the AJE website ([http://learn.aje.com/plos/](about:blank)) for a 15% discount off AJE services. To take advantage of our partnership with Editage, visit the Editage website (www.editage.com) and enter referral code PLOSEDIT for a 15% discount off Editage services.  If the PLOS editorial team finds any language issues in text that either AJE or Editage has edited, the service provider will re-edit the text for free.

Upon resubmission, please provide the following:

a) The name of the colleague or the details of the professional service that edited your manuscript

b) A copy of your manuscript showing your changes by either highlighting them or using track changes (uploaded as a *supporting information* file)

c) A clean copy of the edited manuscript (uploaded as the new *manuscript* file)

Answer: The manuscript was proofread by a professional medical translator (Sarah Chalmers; https://www.medi-translate.com/)

3. We note that you have indicated that data from this study are available upon request. PLOS only allows data to be available upon request if there are legal or ethical restrictions on sharing data publicly. For information on unacceptable data access restrictions, please see <http://journals.plos.org/plosone/s/data-availability#loc-unacceptable-data-access-restrictions>.

In your revised cover letter, please address the following prompts:

a) If there are ethical or legal restrictions on sharing a de-identified data set, please explain them in detail (e.g., data contain potentially identifying or sensitive patient information) and who has imposed them (e.g., an ethics committee). Please also provide contact information for a data access committee, ethics committee, or other institutional body to which data requests may be sent.

b) If there are no restrictions, please upload the minimal anonymized data set necessary to replicate your study findings as either Supporting Information files or to a stable, public repository and provide us with the relevant URLs, DOIs, or accession numbers. Please see <http://www.bmj.com/content/340/bmj.c181.long> for guidelines on how to de-identify and prepare clinical data for publication. For a list of acceptable repositories, please see <http://journals.plos.org/plosone/s/data-availability#loc-recommended-repositories>.

We will update your Data Availability statement on your behalf to reflect the information you provide.

Answer: The data cannot be shared publicly because of ethical restrictions and data protection issues as our dataset includes potentially identifying information.

4. Please ensure that you refer to Figure 1 in your text as, if accepted, production will need this reference to link the reader to the figure.

Answer: A reference to Figure 1 is now included (line 208).

*“As illustrated in Fig 1, the comparison of our results with data from Nolting et al. [8] revealed statistically significant differences (p < 0.05) for the following factors: versatility (3.6 vs. 3.8), completeness of task (3.5 vs. 3.6), scope of action (3.4 vs. 3.8), social support (4.0 vs. 3.7), cooperation (3.6 vs. 3.4), qualitative work demands (2.2 vs. 2.1), works disruptions (2.7 vs. 2.4), information and participation (3.6 vs. 3.3), and benefits (2.9 vs. 2.4).”*

5. We note you have included a table to which you do not refer in the text of your manuscript. Please ensure that you refer to Table 4 in your text; if accepted, production will need this reference to link the reader to the Table.

Answer: A reference to Table 4 is now included (line 221).

*“Table 4 shows a comparison of PrAs in our study population (from 2014) and the comparative study population (from 2000).”*

[Note: HTML markup is below. Please do not edit.]

**Reviewers' comments:**

Reviewer's Responses to Questions

**Comments to the Author**

1. Is the manuscript technically sound, and do the data support the conclusions?

The manuscript must describe a technically sound piece of scientific research with data that supports the conclusions. Experiments must have been conducted rigorously, with appropriate controls, replication, and sample sizes. The conclusions must be drawn appropriately based on the data presented.

Reviewer #1: Yes

2. Has the statistical analysis been performed appropriately and rigorously?

Reviewer #1: Yes

3. Have the authors made all data underlying the findings in their manuscript fully available?

The [PLOS Data policy](http://www.plosone.org/static/policies.action#sharing) requires authors to make all data underlying the findings described in their manuscript fully available without restriction, with rare exception (please refer to the Data Availability Statement in the manuscript PDF file). The data should be provided as part of the manuscript or its supporting information, or deposited to a public repository. For example, in addition to summary statistics, the data points behind means, medians and variance measures should be available. If there are restrictions on publicly sharing data—e.g. participant privacy or use of data from a third party—those must be specified.

Reviewer #1: No

Answer: The data cannot be shared publicly because of ethical restrictions and data protection issues as our dataset includes potentially identifying information.

4. Is the manuscript presented in an intelligible fashion and written in standard English?

PLOS ONE does not copyedit accepted manuscripts, so the language in submitted articles must be clear, correct, and unambiguous. Any typographical or grammatical errors should be corrected at revision, so please note any specific errors here.

Reviewer #1: No

Answer: The manuscript was proofread by a certified medical translator.

5. Review Comments to the Author

Please use the space provided to explain your answers to the questions above. You may also include additional comments for the author, including concerns about dual publication, research ethics, or publication ethics. (Please upload your review as an attachment if it exceeds 20,000 characters)

Reviewer #1:

1. The manuscript is full of typography errors; punctuations.
Answer: The manuscript was proofread by a professional medical translator.

2. Language is main problem
Answer: The manuscript was proofread by a professional medical translator.

3. Not consistent throughout the document
Answer: The manuscript was proofread by a professional medical translator.

4. Don’t use abbreviation in the abstract part
Answer: This was corrected.

5. In the background part there are incomplete sentences
Answer: This was corrected.

6. In tables the decimal places should be consistent
Answer: This was corrected in Tables 1, 2,3 and 4.

7. In the table reporting missing value is not necessary
Answer: This was corrected in Tables 1 and 2.

6. PLOS authors have the option to publish the peer review history of their article ([what does this mean?](https://journals.plos.org/plosone/s/editorial-and-peer-review-process#loc-peer-review-history)). If published, this will include your full peer review and any attached files.


If you choose “no”, your identity will remain anonymous but your review may still be made public.


**Do you want your identity to be public for this peer review?** For information about this choice, including consent withdrawal, please see our [Privacy Policy](https://www.plos.org/privacy-policy).

Reviewer #1: No

**Answer to reviewer comment concerning response rate**

Reviewer comment: “Statistically how it can be generalized with around 30% non-response rate?

Answer: The argument in our sentence was incorrect. The total study had a response rate of 70% of practices. Within the practices, nearly all physicians and practice assistants participated indicating a high interest in the topic.

The text was revised to: It is a strength of our study that it was based on a data set with a large number of participants (550 PrAs). Also, prior analyses had shown that the practice network from which this sample was taken is representative for German primary care practices.
