# Supplementary material for: Practice assistants´ perceived mental workload: A cross-sectional study with 550 German participants addressing work content, stressors, resources, and organizational structure
Source: PLoS One. 2020 Oct 1;15(10):e0240052. doi: 10.1371/journal.pone.0240052 (PMC7529273; doi:10.1371/journal.pone.0240052)
Supplement: Attachment — Submitted filename: Response_to_Reviewers.docx [file pone.0240052.s003.docx]

**Response to Editor**

PONE-D-20-07803R1
Practice assistants´ perceived mental work load: A cross-sectional study with 550 German participants addressing work content, stressors, resources, and organizational structure
PLOS ONE

*Dear Dr. Useche,*

*Please find our revision and answers regarding the additional comments of reviewer 1. We hope that the revision made will adequately address the open points.*

*Best regards,*

*Jan Hoffmann*

Reviewers´ comments:

1. The manuscript has typography errors which are highlighted in the pdf file.

**Answer**: Thank you for highlighting the typography errors. We corrected them accordingly.

2. On the result section of the manuscript I have a great concern with the response rate which is 70.3%. Statistically this will have an impact on the power and it needs clear justification.

**Answer**: We performed a power calculation using the software G-Power 3.1 for both group comparisons to show appropriate sample sizes.

Line 176-178:

*“Power calculations were performed using the software G-Power 3.1 to determine the appropriateness of sample sizes used in the group comparisons.”*

Line 206-208:

*“The power calculation revealed that the sample sizes compared (n=550 versus n=8.121) were sufficient to achieve 80% power to detect small effect sizes of d = 0.12. In the case of greater differences, the power achieved was even higher.”*

Line 226-228:

*“The power calculation revealed that the sample sizes compared (n=550 versus n=324) were sufficient to achieve 80% power to detect small effect sizes of d = 0.2. In the case of greater differences, the power achieved was even higher.”*

Considering our study population, we also feel very comfortable with a response rate of 70%. Struggles to achieve high response rates in similar populations are reported elsewhere:

Redaèlli M, Bassüner S, Teschner D, Stock S. Practice nurses can do more: Online surveys of VERAH-graduates and practice owners. Zeitschrift für Allgemeinmedizin. 2014; 90: 517–22. doi: 10.3238/zfa.2014.0517-0522.

Mergenthal K, Beyer M, Güthlin C, Gerlach FM. Evaluation des VERAH-Einsatzes in der Hausarztzentrierten Versorgung in Baden-Württemberg. Zeitschrift für Evidenz, Fortbildung und Qualität im Gesundheitswesen. 2013; 107:386–93. doi: 10.1016/j.zefq.2013.07.003 PMID: 24075680.

3. Table 1 and 2 some continuous variables are presented in the table as mean and median, which is not acceptable, please remove all continuous variables (age, work experiences, Number of Number of physicians in practice, and Number of PrAs in practice) from the table and narrate the findings.

**Answer**: We do not see a contradiction in mixing continuous variables and categorical variables in Tables 1 and 2. For clarification, we would like to maintain these variables in the table as has been done in other PLOS ONE publications on cross-sectional studies (e.g., Dubois J, Bill A-S, Pasquier J, Keberle S, Burnand B, Rodondi P-Y. Characteristics of complementary medicine therapists in Switzerland: A cross-sectional study. PLoS ONE. 2019; 14:e0224098. doi: 10.1371/journal.pone.0224098 PMID: 31644559)

4. With regard to missing data; it needs clear justification on, how did you manage missing data. As you described in table 1 and table 2 you have variable with missing values.

**Answer**: The management of missing data is described in line 161-162:

*“Non-plausible values were recoded as missing values. Missing data were managed by reporting valid percentages only.”*

In Table 1 and 2 variables with missing data are corrected. Now, only valid percentages are reported. For all variables, the number of analyzed answers is stated in brackets for clarification.

5. In the reference section: When you write references like reference number 6: “Wagner A, Rieger MA, Manser T, Sturm H, Hardt J, Martus P, et al. Healthcare professionals'perspectives on working conditions, leadership, and safety climate: a cross-sectional study. BMC Health Serv Res. 2019; 19: 53. doi: 10.1186/s12913-018-3862-7.” You write the journal name in abbreviated form, but on the other reference like reference number 10: “Theorell T, Hammarstrom A, Aronsson G, Traskman Bendz L, Grape T, Hogstedt C, et al. A systematic review including meta-analysis of work environment and depressive symptoms. BMC Public Health. 2015. doi: 10.1186/s12889-015-1954-4.” you write the journal in full. So, your references should be uniform and journal names better to be in full description.

**Answer**:

Thank you for your comment. We checked all references to make sure that they are cited uniformly with full description of journal names.
